# Supplementary material for: AAV6 vectors provide superior gene transfer compared to AAV9 vectors following intramyocardial administration
Source: Mol Ther Methods Clin Dev. 2025 Jul 15;33(3):101532. doi: 10.1016/j.omtm.2025.101532 (PMC12329528; doi:10.1016/j.omtm.2025.101532)
Supplement: Document S1. Figures S1–S5 [file mmc1.pdf]

OMTM, Volume *33*

Supplemental information

AAV6 vectors provide superior gene transfer compared to AAV9 vectors following intramyocardial administration

Jianan Wang (王嘉南), Timo Jonker, Aina Cervera-Barea, Zhenyu Dong, Ruud N. Visser, Evelien E. Birza, Arie R. Boender, Mischa Klerk, Yuting Yang (杨玉婷), Joyce Visser, Marlijn S. Jansen, Tom C. Grootswagers, Cindy I. Bart, Saskia C.A. de Jager, Silke Schrödel, Christian Thirion, Osne F. Kirzner, Hanno L. Tan, Antoine A.F. de Vries, Joost P.G. Sluijter, Klaus Neef, Joris R. de Groot, Vincent M. Christoffels, and Gerard J.J. Boink

Supplemental information

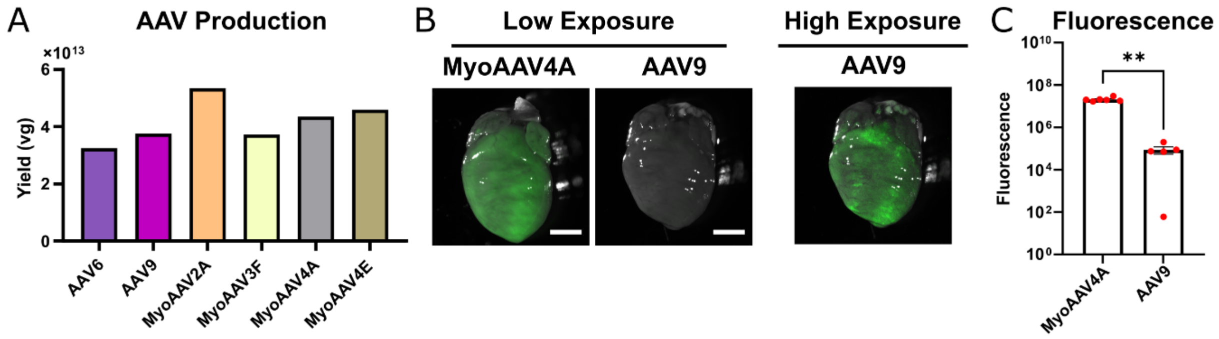

Figure S1. The production and validation of AAV vectors.

(**A**) Yields are similar among different AAV vector pseudotypes. (**B**) Typical heart images of female FVB mice intravenously injected with 2 × 10^{12} vg AAV9 or MyoAAV4A. Scale bar = 2 mm. (**C**) Quantification of the direct GFP fluorescence in mouse hearts, n =5-6. Data are presented as mean ± SEM. Data were compared using Student's t-test. ***P* < 0.01. **; denotes comparison between MyoAAV4A and AAV9.

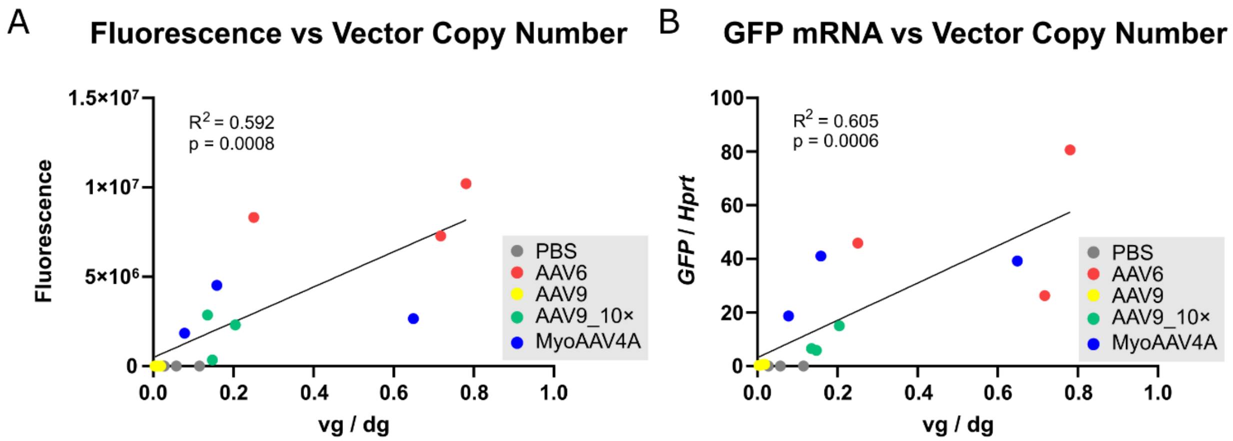

Figure S2. GFP expression correlates to AAV transduction in heart of mice intramyocardially injected with AAV-cTnT-GFP vectors.

(**A**) Correlation of GFP fluorescence and AAV vector copy number, n = 3. (**B**) Correlation of GFP mRNA expression level and AAV vector copy number, n = 3. Linear regression is shown in solid black lines. vg/dg, vector genomes per diploid genome.

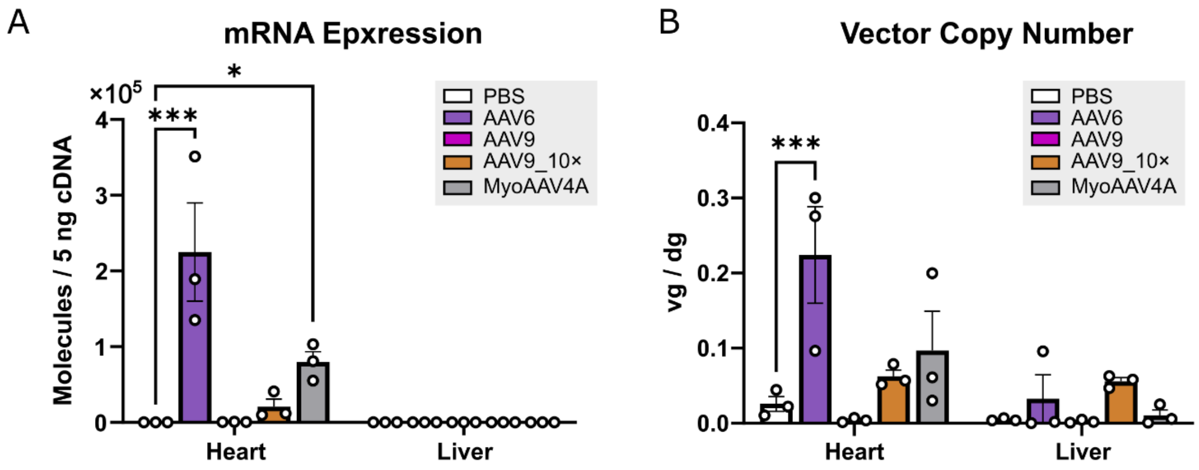

Figure S3. Intramyocardial injection of AAV-cTnT-GFP in mice does not lead to detectable transgene expression in the liver.

(**A**) mRNA expression level of GFP in heart and liver of mice intramyocardially injected with AAV-cTnT-GFP vectors, n = 3. (**B**) AAV genome copies in heart and liver of mice injected with AAV-cTnT-GFP vectors, n = 3. Data are presented as mean ± SEM. Data were compared using one-way ANOVA with *post-hoc* Fisher's LSD test. **P* < 0.05; ****P* < 0.001; ns, not significant. *; denotes comparison between groups and PBS. vg/dg, vector genomes per diploid genome.

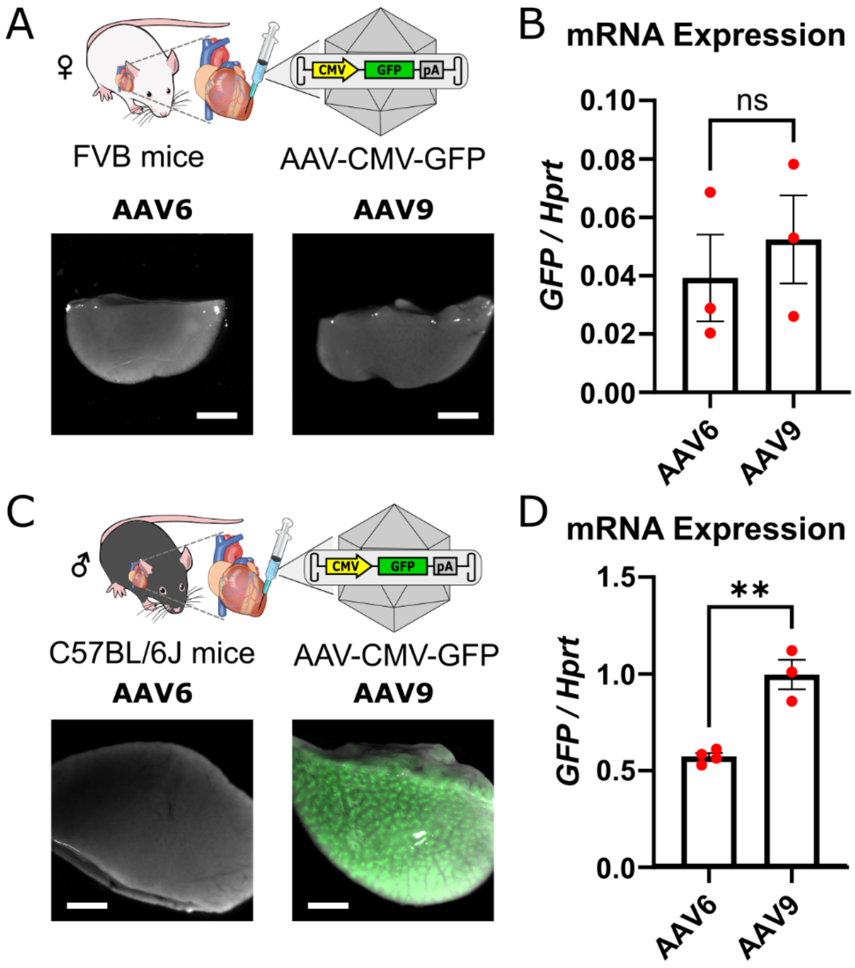

Figure S4. Intramyocardial injection of AAV9-CMV-GFP leads to higher liver expression in C57BL/6J mice than in FVB mice.

(A) Representative liver images of female FVB mice receiving direct intramyocardial injections with GFP-encoding AAV6 or AAV9 pseudotype vectors. Scale bar = 2 mm. (B) Quantification of GFP mRNA expression in the liver of female FVB mice, n = 3. (C) Typical liver images of male C57BL/B6J mice receiving direct intramyocardial injections with GFP-encoding AAV6 or AAV9 pseudotype vectors. Scale bar = 2 mm. (D) Quantification of GFP mRNA expression in the liver of male C57BL/6 mice, n = 3. Data are presented as mean ± SEM. Data were compared using Student's t-test. **P < 0.01; ns, not significant. **; denotes comparison between AAV6 and AAV9.

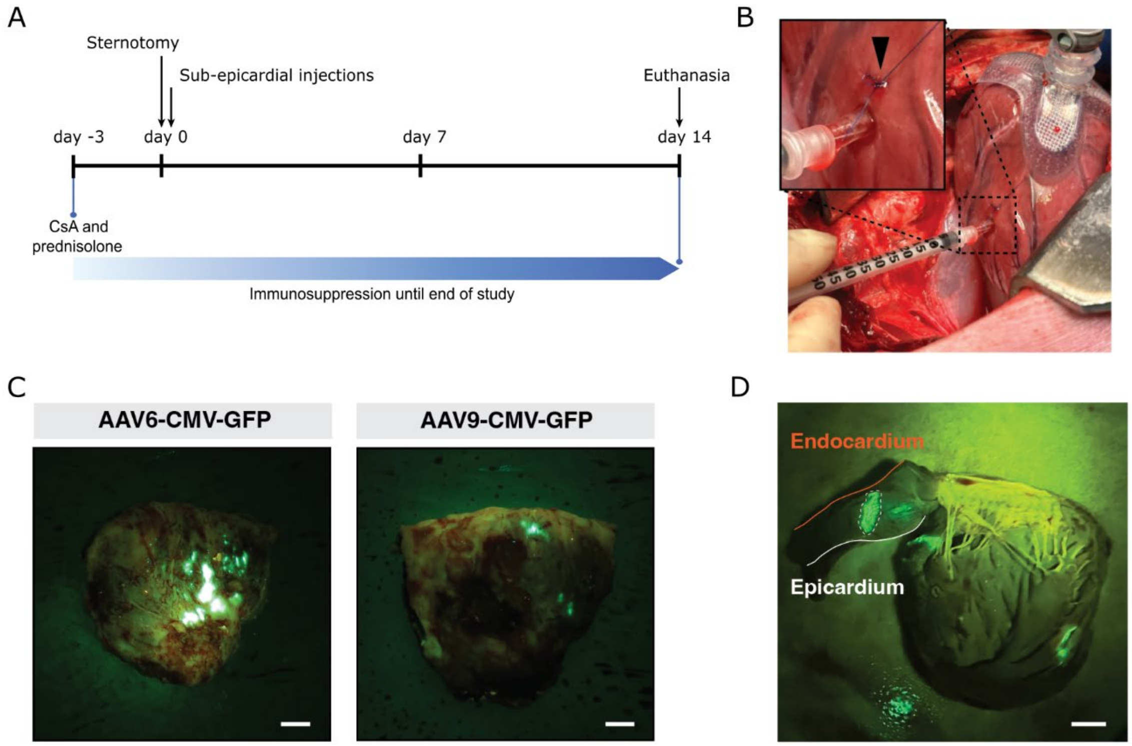

Figure S5. AAV vector-mediated GFP transduction in immunosuppressed pigs.

(A) Study timeline. (B) Heart stabilization from the apex using a heart positioner (Starfish™) to facilitate marking of the injection region with a suture (black arrow). (C) Epicardial view of GFP fluorescent areas from dissected left ventricles, 14 days post-transduction. Scale bar = 1 cm. (D) Endocardial dissection of the left ventricle of an AAV6-CMV-GFP-transduced pig displaying transmural transduction (white dashed oval). Scale bar = 1 cm.
