# Supplementary material for: AAV6 vectors provide superior gene transfer compared to AAV9 vectors following intramyocardial administration
Source: Mol Ther Methods Clin Dev. 2025 Jul 15;33(3):101532. doi: 10.1016/j.omtm.2025.101532 (PMC12329528; doi:10.1016/j.omtm.2025.101532)
Supplement: Document S2. Article plus supplemental information [file mmc2.pdf]

Molecular Therapy
Methods & Clinical Development
Original Article

AAV6 vectors provide superior gene transfer compared to AAV9 vectors following intramyocardial administration

Jianan Wang (王嘉南),[1,11] Timo Jonker,[1,11] Aina Cervera-Barea,[1,2,3,11] Zhenyu Dong,[4,5] Ruud N. Visser,[1] Evelien E. Birza,[1,2,3] Arie R. Boender,[1,6] Mischa Klerk,[1] Yuting Yang (杨玉婷),[6] Joyce Visser,[2] Marlijn S. Jansen,[2] Tom C. Grootswagers,[1] Cindy I. Bart,[7] Saskia C.A. de Jager,[2] Silke Schrödel,[8] Christian Thirion,[8] Osne F. Kirzner,[6,9] Hanno L. Tan,[4,6,10] Antoine A.F. de Vries,[7] Joost P.G. Sluijter,[2,3] Klaus Neef,[6,10] Joris R. de Groot,[4,5] Vincent M. Christoffels,[1] and Gerard J.J. Boink[1,4,5,6]

[1]Department of Medical Biology, Amsterdam Cardiovascular Sciences, Amsterdam University Medical Centers, University of Amsterdam, 1105 AZ Amsterdam, the Netherlands; [2]Laboratory for Experimental Cardiology, Regenerative Medicine Center Utrecht, Circulatory Health Research Center, University Medical Center Utrecht, Utrecht University, 3584 CX Utrecht, the Netherlands; [3]Department of Cardiology, Laboratory of Experimental Cardiology, Division Heart and Lungs, University Medical Center Utrecht, 3584 CX Utrecht, the Netherlands; [4]Department of Experimental Cardiology, Amsterdam Cardiovascular Sciences, Amsterdam University Medical Centers, University of Amsterdam, 1105 AZ Amsterdam, the Netherlands; [5]Department of Clinical Cardiology, Amsterdam Cardiovascular Sciences, Amsterdam University Medical Centers, University of Amsterdam, 1105 AZ Amsterdam, the Netherlands; [6]PacingCure BV, 1018 WB Amsterdam, the Netherlands; [7]Laboratory of Experimental Cardiology, Department of Cardiology, Leiden University Medical Center, Leiden 2300 RC, the Netherlands; [8]Revvity Gene Delivery GmbH, 82166 Gräfelfing, Germany; [9]Department of Anesthesiology, Amsterdam University Medical Center, 1081HV Amsterdam, the Netherlands; [10]Netherlands Heart Institute, 3511 EP Utrecht, the Netherlands

Cardiac gene therapy using adeno-associated viral (AAV) vectors holds great promise for treating heart diseases but would benefit from more potent AAV vectors. Vectors based on the AAV serotypes 6 and 9 have been used in pre-clinical gene therapy studies, yet the therapeutic outcomes varied depending on the experimental model and delivery route used. Here, we evaluated the transduction efficiency of AAV6, AAV9, and AAV9-derived MyoAAVs for local cardiac delivery. Vectors were tested in neonatal rat ventricular myocytes, and subsequently in mouse hearts by direct intramyocardial injection. Vector genome levels, mRNA expression levels, and fluorescence were measured. The AAV6 and AAV9 vectors were further validated in porcine hearts, human-induced pluripotent stem-cell-derived cardiomyocytes, and human atrial myocardial slices. In both rat cardiomyocytes and mouse hearts, AAV6 exhibited the highest transduction efficiency. Direct comparison of the AAV6 and AAV9 vectors in porcine and human models confirmed that AAV6 is more potent. In conclusion, AAV6 vectors are superior to AAV9 and its derivative vectors for cardiac transduction by direct intramyocardial injection. In addition, the *in vivo* transduction efficiency correlates with *in vitro* and *ex vivo* assays, thereby facilitating the development of more potent AAV variants for cardio-selective delivery methods.

INTRODUCTION

Cardiovascular disease remains the top health threat in terms of mortality and morbidity, affecting 30% of the global population.[1] Gene therapy is a promising method to treat diseases by delivering genetic information into target cells, ultimately altering cellular function to improve disease status. Adeno-associated viral (AAV) vectors have become an attractive delivery vehicle for *in vivo* gene delivery purposes due to their ability to achieve long-term transgene expression and their low immunogenicity.[2] To date, more than 350 clinical trials have been carried out employing AAV vectors,[3] and a total of six AAV-based gene therapy products (Glybera, Luxturna, Zolgensma, Roctavian, Hemgenix, and Kebilidi/Upstaza) have received regulatory approval from either the Food and Drug Administration (FDA) or the European Medicines Agency (EMA).[4,5] However, the development of gene therapies for cardiovascular diseases lags behind that of other organ systems. No AAV-based cardiac gene therapy has been approved yet, partially due to the lack of effective AAV gene delivery methods in large mammalian (including human) hearts.[6,7] Failure of the largest cardiac clinical gene therapy trials performed to date, which utilized AAV1 vectors, has largely been attributed to inefficient gene transfer.[8,9] As a result, recent and ongoing clinical trials have shifted toward alternative AAV serotypes in an effort to enhance gene delivery efficacy.[10–15]

Received 3 April 2025; accepted 9 July 2025;
https://doi.org/10.1016/j.omtm.2025.101532.

[11]These authors contributed equally

Correspondence: Gerard J.J. Boink, Department of Medical Biology, Amsterdam Cardiovascular Sciences, Amsterdam University Medical Centers, University of Amsterdam, Amsterdam 1105 AZ, the Netherlands.
E-mail: g.j.boink@amsterdamumc.nl

© 2025 The Author(s). Published by Elsevier Inc. on behalf of The American Society of Gene and Cell Therapy.
This is an open access article under the CC BY license (http://creativecommons.org/licenses/by/4.0/).

Hundreds of AAV serotypes have been discovered in humans, non-human primates, and various other species, displaying different tissue and cell type tropisms.[16] Among these serotypes, AAV1, AAV6, AAV8, AAV9, AAVrh10, and AAVrh74 have been demonstrated to be cardiotropic in different settings.[17–23] Among these cardiotropic serotypes, AAV6 and AAV9 are the most widely used and arguably the most potent for cardiac gene transfer.[24–28] In various *in vitro* and *ex vivo* models, including neonatal rat ventricular cardiomyocytes (NRVMs), rat and pig cardiac slices, human embryonic stem-cell-derived cardiomyocytes, human-induced pluripotent stem-cell-derived cardiomyocytes (hiPSC-CMs), and human myocardial slices, AAV6 outperformed AAV9.[29,30] On the other hand, in several other comparative cardiac gene therapy studies, AAV9 performed equally well as, or even outperformed, AAV6.[3,24,30–33] This led to the notion that AAV transduction properties *in vitro*/*ex vivo* may differ from those *in vivo*. Importantly, most *in vivo* studies were done after intravenous vector delivery, which may not translate well to other routes of vector administration.

Intramyocardial injection represents a highly attractive method for attaining high transduction efficiency within a defined target region. It is particularly useful in gene-therapy-mediated biological pacemakers, where focal delivery of the vectors is critical to create a local pacemaker substrate while leaving the rest of the myocardium unaffected.[34] Moreover, this mode of gene delivery is also considered for the treatment of myocardial infarction and scarring, aiming to promote local tissue regeneration[35–38] or prevent ventricular arrhythmias,[39,40] which in case of the former is now progressing toward the first-in-human clinical trial testing.[10] Another advantage of intramyocardial injection is the reduced exposure in the bloodstream, which significantly minimizes the risk of neutralization by circulating antibodies.[41] Intramyocardial injection can be performed either surgically or percutaneously.[42,43] The surgical approach, involving open-heart surgery, offers high precision and flexibility in vector delivery but is invasive. In contrast, the percutaneous approach uses catheter-based transendocardial injection, making it minimally invasive and thus more favorable in clinical settings.

Unlike studies employing intravenous injection, comparative studies using intramyocardial injection are limited and inconsistent. In these studies employing intramyocardial injection, AAV6 was reported the most potent in pigs, dogs, and non-human primates, suggesting it is superior to AAV9.[35,44,45] In contrast, a mouse study showed AAV6 to be inferior to AAV9 in cardiac gene transfer upon intramyocardial injection.[46] Moreover, intramyocardial injection of AAV1-8 vectors in rats showed AAV8 to be better than AAV6,[47] suggesting a suboptimal performance of AAV6 in cardiac gene transfer. Such profound difference in AAV serotype performance between different models complicates the development of more potent AAV variants. It potentially also contributed to the selection of suboptimal serotypes and the application of unnecessarily high vector dosages for pre-clinical and clinical studies.[20,38,48–53] Ultimately, such suboptimal serotypes require (much) higher dosages, leading to increased risk of side effects and immune activation as well as higher production costs.

Our present work systematically evaluates the performance of different AAV serotypes in terms of cardiac transgene expression and transduction efficiency utilizing various *in vitro*, *in vivo*, and *ex vivo* models. Our results indicate that, for AAV vector-mediated gene transfer to cardiomyocytes, *in vitro* and *ex vivo* results obtained with a particular AAV vector are highly predictive for the *in vivo* transduction efficiency via direct injection and that the performance of AAV vectors in different species is consistent. Moreover, these experiments confirm the potency of AAV6 in cardiac gene transfer across different species, including mouse, pig, and human, particularly in the setting of direct intramyocardial injection.

RESULTS

AAV6 and MyoAAV4A achieve high transgene expression in NRVMs

We started by producing six different AAV vectors carrying a chicken *Tnnt2* (cTnT) promoter-driven enhanced green fluorescent protein (GFP) transgene (Figure 1A) and pseudotyped with capsids of two natural cardiotropic AAV serotypes (i.e., AAV6 and AAV9) and four engineered AAV9 capsids selected for high muscular transduction (MyoAAV2A, -3F, -4A, and -4E).[26] The production yields of these vectors were similar, ranging from 3.3 to 5.3 $\times$ 10^{13} vector genomes (vg) (Figure S1A). To validate the performance of the engineered AAV capsids, we injected mice intravenously with MyoAAV4A and AAV9 and found that, similar to previous reports,[26,54] MyoAAV4A led to significantly higher transgene expression compared to AAV9 4 weeks post-injection (Figures S1B and S1C).

We subsequently compared the transgene expression *in vitro* in NRVMs transduced with all of the above-mentioned AAV vectors at an MOI of 10^4 vg/cell and incubated for 3 days until flow cytometry and reverse-transcription quantitative PCR (RT-qPCR) analyses (Figures 1A and 1B). Additionally, a 10-fold higher dose for AAV9 (AAV9_10×) was included to explore the effect of differential vector dosing. Flow cytometry revealed that AAV6 leads to the highest transduction efficiency in NRVMs, followed by MyoAAV4A and MyoAAV2A (Figure 1C). AAV9 and AAV9_10× gave the lowest transduction efficiency of all AAV vector pseudotypes, significantly lower than AAV6 (Figure 1C). RT-qPCR analyses revealed a similar pattern as flow cytometry, with AAV6 giving the highest transgene expression, followed by MyoAAV4A and MyoAAV2A (Figure 1D). From these results, we selected the most potent MyoAAV variant MyoAAV4A, as well as AAV6 and AAV9 for further *in vivo* testing.

AAV6 outperforms AAV9 and MyoAAV4A in direct intramyocardial-injection-mediated gene transfer in mouse myocardium

Subsequently, we compared the ability of the cTnT-driven AAV6, AAV9, and MyoAAV4A pseudotype vectors to transduce healthy mouse myocardium after direct intramyocardial injection. We injected 10^{11} vg of these vectors into the left ventricular wall of female FVB mice (Figure 2A). An additional high-dose (10^{12} vg) group

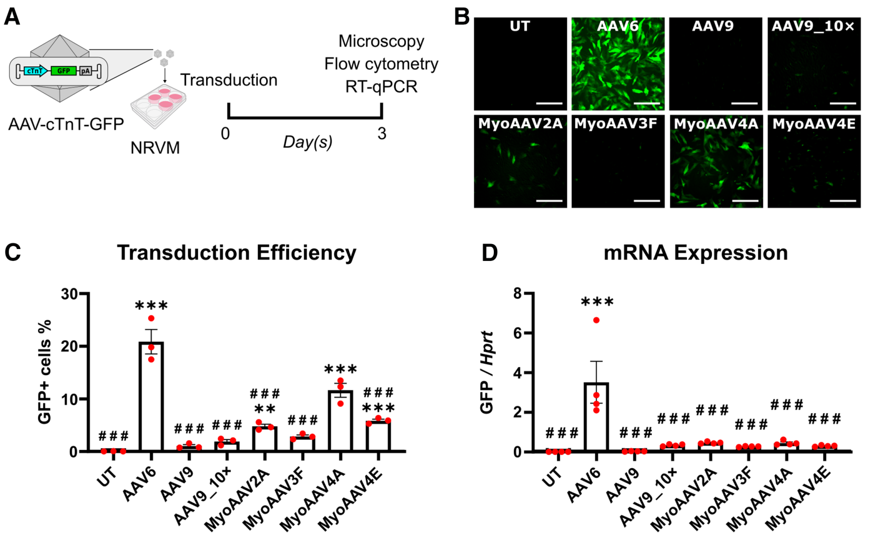

Figure 1. AAV6 and MyoAAV4A efficiently transduce NRVMs

(A) Experimental design. (B) Direct fluorescent images of NRVMs 3 days post-transduction with various AAV vector pseudotypes. Scale bars, 100 μm. (C) Percentage of GFP-expressing cells determined by flow cytometry. (D) Relative expression levels of GFP determined by RT-qPCR. Data are presented as mean ± SEM. Data were compared using one-way ANOVA with post-hoc Fisher's LSD test. $^{**}p < 0.01$; ***, $^{\#\#\#}p < 0.001$. * denotes comparison between groups and UT. # denotes comparison between groups and AAV6. UT, untransduced; AAV9_10×, AAV9 at a 10 times higher dose.

(AAV9_10×) for AAV9 was included. Four weeks later, GFP fluorescence was observed in all AAV-vector-injected hearts using direct fluorescence imaging (Figure 2B), further microscopically confirmed by immunofluorescence staining of GFP at the injection site (Figure 2C). To evaluate transgene expression levels, we utilized macroscopy for fluorescence quantification and RT-qPCR for mRNA quantification. Both methods revealed AAV6 as the most efficient variant for transgene expression, followed by MyoAAV4A (Figures 2D and 2E). Similar to our *in vitro* results (Figures 1C and 1D), AAV9_10× led to significantly higher transgene expression compared to AAV9, but the expression levels still remained significantly lower than those of AAV6 or MyoAAV4A (Figures 2D and 2E). To estimate transduction efficiency, the vector copy number was quantified, showing AAV6 to be the most efficient of the tested AAV vectors (Figure 2F). Both fluorescence and mRNA expression showed significant correlation to vector genome copy number (Figure S2). None of the tested vectors induced hepatic transgene expression, and vector genomes were detected only in the liver of animals receiving the high dose of AAV9 and one AAV6 animal (Figure S3). Our data indicate that AAV6 is more efficient in transducing mouse cardiomyocytes than AAV9 or MyoAAV4A, which is in line with the results in NRVMs (Figure 1).

Intramyocardial injection of AAV vectors in mice does not induce vector-related cardiac fibrosis at 4 weeks post-injection

To assess the potential toxicity of intramyocardial AAV injections, we quantified cardiac fibrosis using histological staining on heart sections from Dulbecco's PBS- and AAV vector-injected mouse hearts (Figure 3). Picrosirius red staining revealed moderately increased collagen levels, indicating mild cardiac fibrosis at the injection site in both PBS- and AAV-treated animals (Figure 3A). No significant differences were observed among the groups including the control PBS group (Figure 3B), suggesting that the fibrosis might stem from the injection procedure itself rather than from the AAV transduction, for example due to needle track trauma or puncture of the epicardium. Additionally, consecutive sections stained for collagen and GFP showed no correlation between the extent of fibrosis and GFP expression (Figure 3C). These results indicate that AAV vector transduction and GFP expression do not increase cardiac fibrosis in hearts following intramyocardial injection at least within the first 4 weeks post-injection.

AAV6 more efficiently transduces mouse myocardium after intramyocardial injection than AAV9 independently of transgene promoter and mouse sex or strain

Our results have shown that AAV6 is more efficient than AAV9 in cardiac gene transfer *in vitro* and *in vivo* upon direct intramyocardial injection (Figures 1 and 2). However, these results were performed exclusively with vectors produced in Amsterdam UMC using the cardiomyocyte-specific cTnT promoter. To test the robustness of our findings, we repeated part of the experiments using AAV vectors carrying a ubiquitous human cytomegalovirus (CMV) immediate-early gene promoter, produced and titrated by Revvity Gene Delivery, and we included mice from different strains and sex (FVB female and C57BL/6J male) (Figure 4), as these variables are all known to potentially impact AAV performance.[55–60]

First, we transduced NRVMs with AAV6 and AAV9 pseudotype vectors in which GFP expression was driven by the CMV promoter and quantified GFP-specific mRNA levels three days post-transduction (Figure 4A). GFP expression was detected by direct fluorescence microscope in both groups (Figure 4B), although AAV6 led to significantly higher transgene expression in NRVMs compared to AAV9 (Figure 4C). These findings are in line with our results obtained with the cTnT-promoter-containing AAV vectors produced in Amsterdam UMC (Figure 2).

We then injected these vectors into the left ventricular free wall of FVB female (as used in Figure 2) and C57BL/6J male mice, which were previously used by Prasad et al.[46] to compare the gene transfer efficiency of different AAV vector pseudotypes following intramyocardial injection. In FVB female mice, AAV6 resulted in a 9.4-fold higher GFP mRNA level than AAV9 while the GFP fluorescence

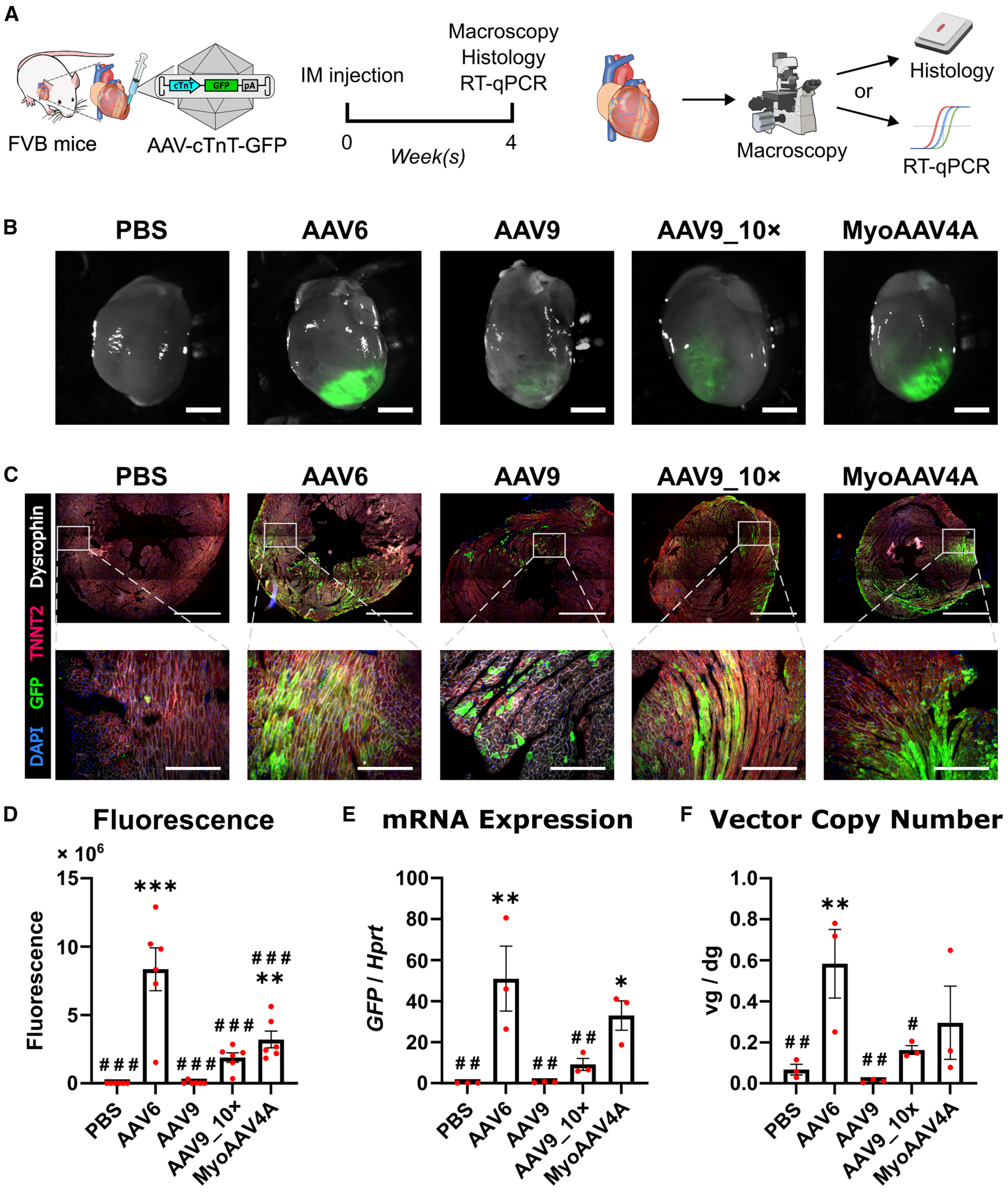

Figure 2. AAV6 and MyoAAV4A efficiently transduce mouse myocardium

(A) Experimental design. (B) Direct fluorescent images of mouse hearts. Scale bars, 2 mm. (C) Immunostaining images of hearts and zoom-in images from the injection sites. Scale bars, 1 mm (upper) or 200 μm (lower). (D) Quantification of the integrated density of direct GFP fluorescence, $n = 6$. (E) mRNA expression level of GFP in left ventricles, $n = 3$. (F) AAV vector genome copies in ventricles, $n = 3$. Data are presented as mean ± SEM. Data were compared using one-way ANOVA with *post-hoc* Fisher's LSD test. *, #$p < 0.05$; **, ##$p < 0.01$; ***, ###$p < 0.001$. * denotes comparison between groups and PBS. # denotes comparison between groups and AAV6. IM, intramyocardial; AAV9_10×, AAV9 at a 10 times higher dose; vg/dg, vector genomes per diploid genome.

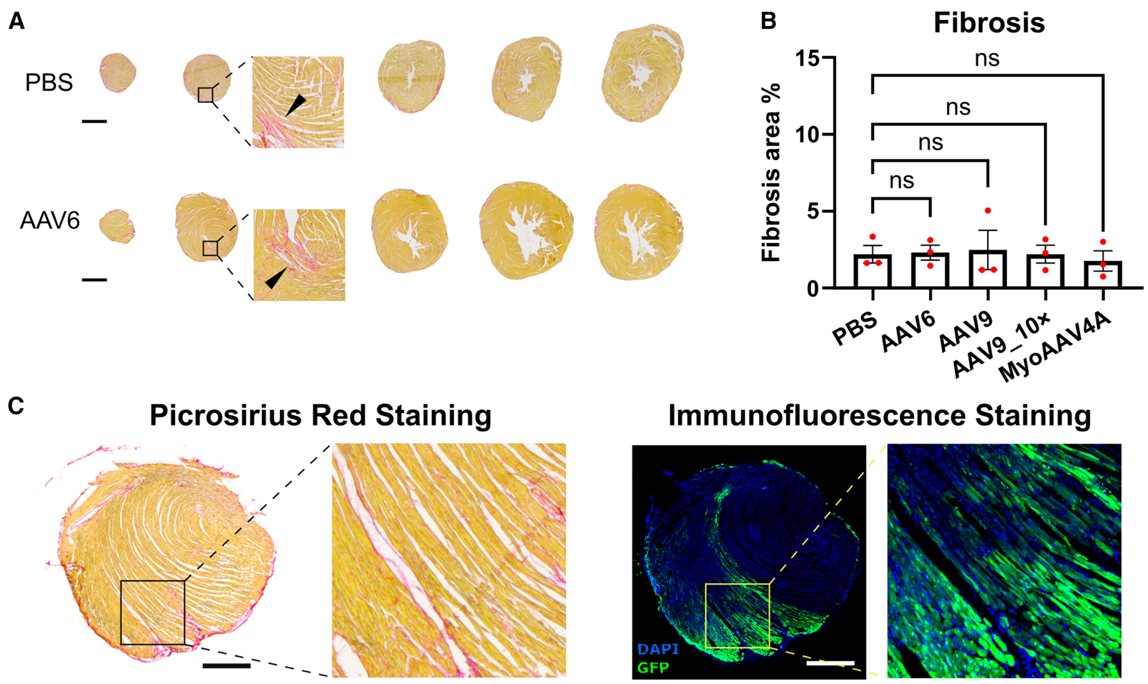

Figure 3. Intramyocardial injection of AAV vector does not induce vector-related cardiac fibrosis

(A) Example of picrosirius red staining images of hearts injected with PBS or AAV6-cTnT-GFP vector. Black arrows indicate regions of cardiac fibrosis. Scale bars, 1 mm. (B) Quantification of the fibrosis area in hearts injected with PBS or AAV vectors. No significant differences were observed among the groups, $n = 3$. (C) Picrosirius red (left) and immunofluorescence (right) staining images of a heart injected with AAV6-cTnT-GFP. Stainings were performed using consecutive sections. Scale bars, 500 μm. Data are presented as mean ± SEM. Data were compared using one-way ANOVA with *post-hoc* Fisher's LSD test. ns, not significant.

($p = 0.11$) and vector copy number ($p = 0.10$) did not significantly differ between the two vectors (Figures 4D–4G). Also in C57BL/6J male mice, AAV6 exhibited a higher transduction efficiency than AAV9 (Figures 4H–4K). We next examined transgene expression in the liver of the animals (Figure S4). Hepatic transgene expression visualized by direct GFP fluorescence was only detected in C57BL/6J males (FiguresS4A–S4C), in accordance with a previous report indicating sex differences in liver transduction.[61] Additionally, AAV9 led to significantly more gene expression and fluorescence than AAV6 (Figures S4C and S4D). Collectively, our data suggest that AAV6 is more efficient than AAV9 in cardiac gene transfer independently of the expression cassette and mouse sex or strain, while also leading to lower hepatic transduction.

AAV6 is more efficient in direct cardiac gene transfer than AAV9 in pig myocardium

To test if there is a species-dependent transduction efficiency, we compared the transduction efficiency of AAV6 and AAV9 in pigs, a relevant translational large animal model for cardiac diseases. Male pigs were injected at low (10^{11} vg in 20 μL) or high dose (10^{12} vg in 200 μL) with CMV-GFP AAV6 and AAV9 vectors that were applied via sub-epicardial injection at two separate sites in the left ventricle (Figure 5A). Two weeks post-injection, hearts were exposed, and injection sites were assessed by macroscopic GFP fluorescence and suture marks (Figure S5). At each injection site, direct GFP fluorescence was detected in the surrounding myocardium (Figure 5B). GFP expression was further confirmed via immunofluorescence microscopy (Figure 5C). RT-qPCR again showed that AAV6 led to significantly higher transgene expression and more vector genomes at the high-dose injection site, when compared to AAV9 (Figures 5D and 5E). Similar trends were observed at the low-dose injection sites (Figures 5D and 5E), although they did not reach statistical significance. These samples exhibited greater variability, likely due to imprecise sampling of the small, transduced area, which resulted in more tissue outside the injection site being included in the analyzed tissue samples. Altogether, these data suggest that AAV6 is more efficient in transducing pig myocardium than AAV9 upon direct intramyocardial injection.

AAV6 outperforms AAV9 in transducing hiPSC-CMs and human myocardial slices

Next, we investigated whether we could recapitulate the findings from the rodent and pig experiments in human translational models. We transduced hiPSC-CMs at MOIs of 2×10^3 and 2×10^4 vg/cell with AAV6 and AAV9 pseudotype vectors carrying the CMV promoter-driven *GFP* transgene (Figure 6A). Five days later, GFP fluorescence was observed in all conditions, and cells were collected for further molecular analyses. RT-qPCR on AAV vector genomes showed a significantly higher transduction efficiency of AAV6 compared to AAV9 at both MOIs (Figure 6B). These results are in

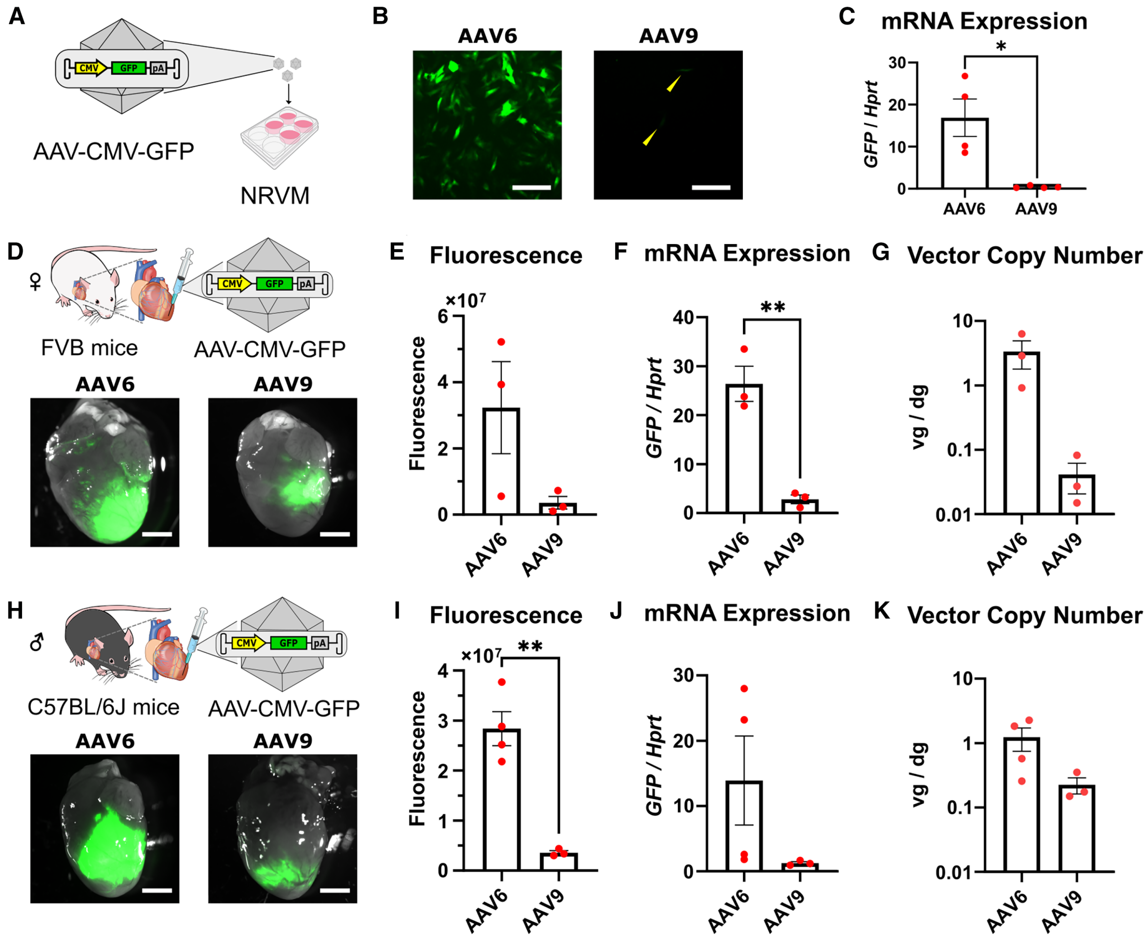

Figure 4. AAV6 outperforms AAV9 independently of the expression cassette and mouse background

(A) Scheme of the *in vitro* experiment. (B) Direct fluorescent images of NRVMs 3 days post-transduction with AAV6- and AAV9-pseudotyped vectors. Yellow arrows indicate weakly GFP-expressing cells. Scale bars, 100 μm. (C) Expression level of GFP mRNA determined by RT-qPCR, $n = 4$. (D) Scheme of the *in vivo* experiment and representative heart images. Scale bars, 2 mm. (E) Quantification of direct GFP fluorescence, (F) GFP mRNA expression, and (G) AAV vector genome copies in FVB female mice injected with AAV6- or AAV9-CMV-GFP, $n = 3$. (H) Scheme of the *in vivo* experiment and representative heart images. Scale bars, 2 mm. (I) Quantification of direct GFP fluorescence, (J) GFP mRNA expression, and (K) AAV vector genome copies in C57BL/6J male mice injected with AAV6- or AAV9-CMV-GFP, $n = 4$. Data are presented as mean ± SEM. Data were compared using Student's t test. $*p < 0.05$; $**p < 0.01$. * denotes comparison between AAV6 and AAV9. vg/dg, vector genomes per diploid genome.

accordance with previous reports.[29,62] We continued our study with an established *ex vivo* human left atrial appendage slices (LAAS) model. Human LAASs were transduced with 10^{11} vg vectors and incubated for 3 days before molecular analyses (Figure 6C). RT-qPCR on AAV vector genomes revealed that AAV6 more efficiently transduced the LAASs as compared to AAV9 (Figure 6D). Collectively, these data suggest that AAV6 outperforms AAV9 in transducing human myocardium.

DISCUSSION

Our data indicate that AAV6 pseudotyped vectors outperform AAV9 pseudotyped vectors, a benchmark in the cardiac gene therapy field, and AAV9-derived engineered variants, regarding cardiac transduction efficiency following intramyocardial injection.

Both AAV6 and AAV9 have been successfully utilized in cardiac gene therapy studies via intramyocardial injection, showing reasonable transduction efficiencies.[35,38] However, identifying more potent AAV vectors can further aid in achieving clinically relevant transduction levels with acceptable vector doses and help reduce inter-individual variability in cardiac transduction efficiencies.[6,7] In studies comparing AAV6 and AAV9 through intramyocardial injections, results have varied between small (mouse) and large animals (pig, dog, and non-human primates), sometimes showing conflicting outcomes.[44–46] In our study, we compared both vectors in cultures of rat and human cardiomyocytes, human atrial tissue slices, and mouse and pig hearts. We did not observe such discrepancies in our experiments, as AAV6 consistently outperformed AAV9 in terms of transgene expression and transduction efficiency. We also assessed the potency of AAV vectors in mice

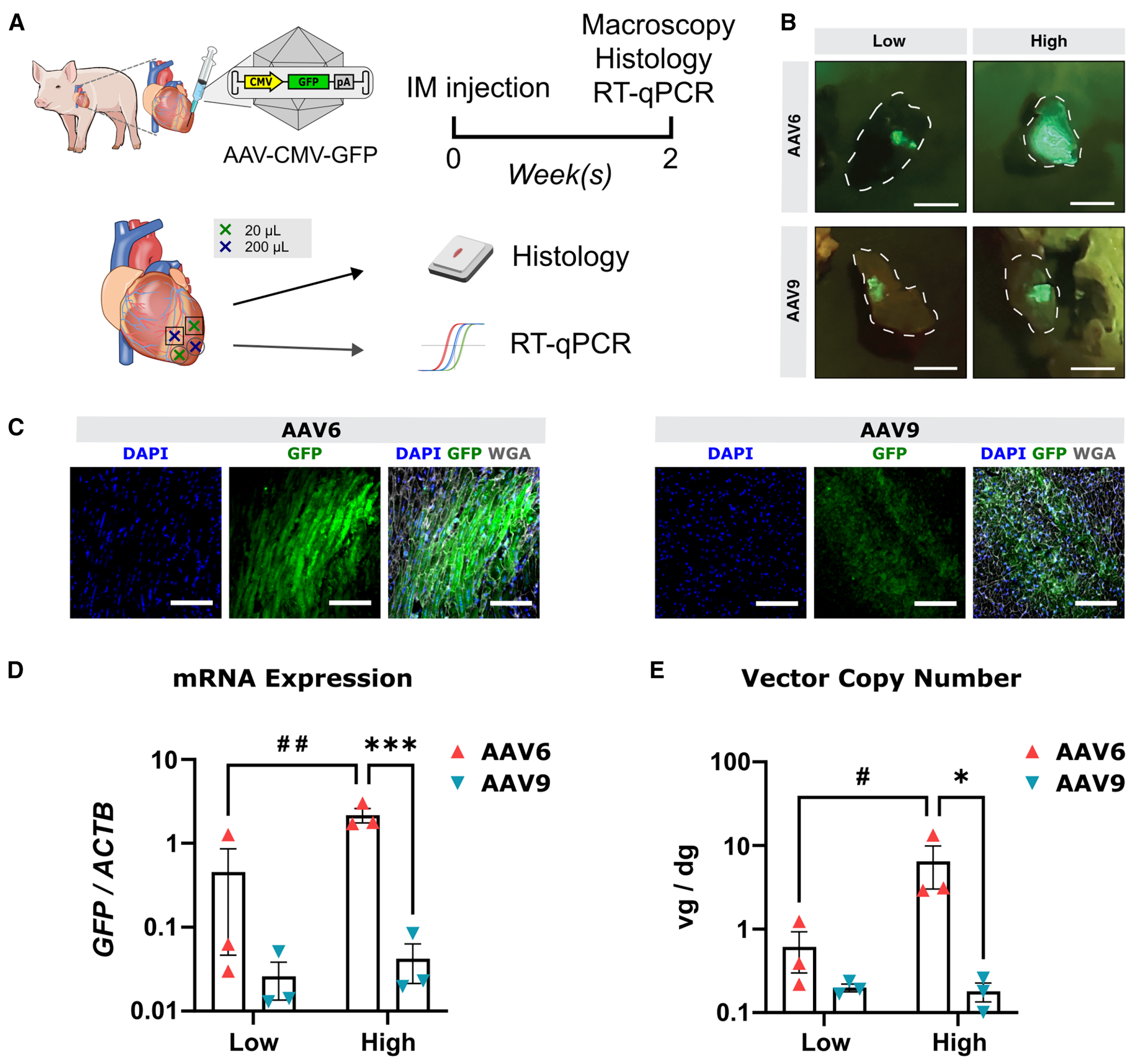

Figure 5. AAV6 outperforms AAV9 in transducing pig myocardium

(A) Experimental design. (B) Direct fluorescent macroscopy of pig heart tissues injected with AAV6- or AAV9-CMV-GFP. Sampled tissues are encircled using a white dashed line. Scale bars, 1 cm. (C) Immunostaining images from pig heart tissues injected with 200 µL (high dose) AAV6- or AAV9-CMV-GFP. Scale bars, 200 µm. (D) Quantification of the GFP mRNA expression and (E) AAV vector genome copies in pig heart tissues injected with AAV6- or AAV9-CMV-GFP, $n = 3$. Data are presented as mean ± SEM. Data were compared using two-way ANOVA with post-hoc Fisher's LSD test. *,# $p < 0.05$; ## $p < 0.01$; *** $p < 0.001$. * denotes comparison between AAV serotypes. # denotes comparison between injection volumes. IM, intramyocardial. vg/dg, vector genomes per diploid genome.

using two independent vector sources and two mouse strains and used different durations of transduction for *in vitro*, mouse and pig experiments to further validate the robustness of our findings. One explanation for the previously reported discrepancy between small and large animals is the use of a relatively large needle (29 G) in the mouse study.[46] This could generate more vector leakage into the circulation, for example by vessel puncture or back-flush, resulting in a different transduction environment where vectors that are more potent in the context of systemic administration, such as AAV9, may perform better.[24,31,32] In support of this hypothesis, Prasad et al. reported significantly higher transgene expression (up to 35-fold) in liver compared to controls, despite the use of the cardiomyocyte-specific cTnT promoter,[46] while we detected none when injecting vector with the same promoter using Hamilton syringes (31-G needles). As a result, our data highlight the robust consistency across different models and are very encouraging with regard to eventual application in human patients, proposing AAV6 as an efficient delivery vehicle for cardiac gene transfer in the context of cardio-selective vector delivery methods such as direct intramyocardial injection and likely also other local methods such as epicardial painting and intracoronary infusion.[63–66]

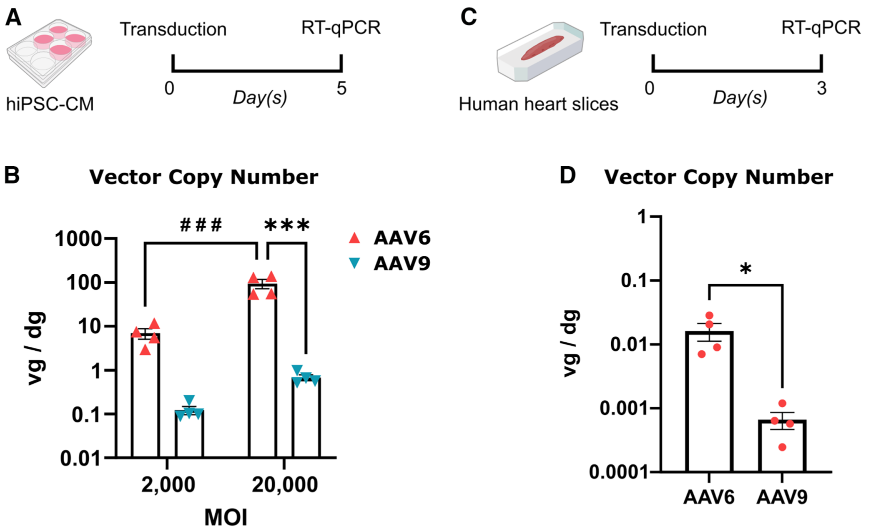

Figure 6. AAV6 outperforms AAV9 in transducing hiPSC-CMs and human heart slices

(A) Scheme of the hiPSC-CM experiment. (B) Quantification of the AAV vector genomes in hiPSC-CMs transduced with AAV6- or AAV9-CMV-GFP, $n = 4$. (C) Scheme of the human LAAS experiment. (D) Quantification of the AAV vector genomes in human LAASs transduced with AAV6- or AAV9-CMV-GFP, $n = 4$ donors. Data are presented as mean ± SEM. Data were compared using two-way ANOVA with *post-hoc* Fisher's LSD test (B) or Student's t test (D). $*p < 0.05$; ***, ###$p < 0.001$. * denotes comparison between AAV serotypes. # denotes comparison between multiplicities of infection. MOI, multiplicity of infection. vg/dg, vector genomes per diploid genome.

Although AAV9 has a lower transduction efficiency than AAV6, it still performed reasonably well in our work, particularly when combined with the strong CMV promoter, which may explain the successful transduction observed in several studies using AAV9.[38,49,51,53] Additionally, AAV9 is currently being used in multiple clinical trials, utilizing intramyocardial, intracoronary, and intravenous injection methods[10–14] and remains a promising tool for cardiac gene therapy. However, for applications requiring a high transduction efficiency, such as those based on dual AAV vectors or those requiring correction of a genetic defect in the majority of *in vivo* cardiomyocytes, AAV9 might not provide the required efficiency, potentially compromising functional outcomes. While administering higher doses can increase transduction efficiency, it also leads to more off-target transduction, as shown in our *in vivo* experiments. Additionally, excessively high doses may raise safety concerns, as vector doses correlate to vector immunogenicity in clinical trials,[67] and high doses reportedly influenced the contractility performance of cardiac slices.[68]

In our NRVM and mouse experiments, we included AAV variants named MyoAAVs. These variants were generated by directed evolution of AAV9 toward muscular tropism via intravenous injection and exhibited stronger binding to cardiomyocytes through their RGD motifs.[26] To our knowledge, we are the first group to study the transduction efficiency of these variants in the context of intramyocardial injection. In mouse hearts, MyoAAV4A led to significantly higher transgene expression and transduction efficiency compared to its parental serotype AAV9 when intramyocardially injected. Despite not outperforming AAV6, it led to high transgene expression and could serve as a reasonable alternative to AAV6.

Dosing plays a critical role in gene therapy, as it influences both transduction efficiency and the immune response. The dose we used in mice was 10^{11} vg/mouse (except for AAV9_10× group), which is around 2- to 10-fold higher than the previous mouse experiments.[46,48,49] This dose achieved high transgene expression in a large region of the heart in case of AAV6. However, direct comparisons of expression level and spread between this and previous studies are not possible due to the different sampling and detection methods. The dose we used in pigs was 10^{12} vg per injection for the 200 μL injection site, which matches the dose reported by Gabisonia et al.[35] The transduction efficiency, as measured by viral genome copies, was also comparable to their findings.[35] At this dose, AAV6 administration resulted in 6.4 vg/dg at the injection site, indicating a high level of transduction. Multiple injections may be required to adequately cover the desired region but AAV6 at this dose appears to be sufficient to achieve effective transduction for each individual injection site. In this study, we used immunosuppressed pigs, and it is possible that in immunocompetent pigs the high local transduction evokes immune responses. It has been reported that intramyocardial injection of AAV2 expressing TNFRII-Fc at 5×10^{11} vg per injection site (5×10^{12} vg in total) triggered immune responses while the same dose of empty AAV2 did not.[69] This suggests that the immune response does not solely depend on the vector dose. Indeed, while the magnitude of the immune responses depends on the vector dose,[67] it is also affected by several other factors.[70] Therefore, immune responses must be carefully evaluated for each specific gene therapy, taking these variables into account.

To date, AAV engineering/screening efforts aiming at improving cardiac transduction have used intravenous injection.[26,71,72] However, these attempts always result in not only capsids with stronger binding properties to cardiomyocytes but also those with lower liver binding or better endothelial passage,[71,73,74] making it difficult to translate results to locally administered applications.[75] Local vector application, such as intramyocardial injection, intracoronary infusion, or epicardial painting, differs from intravenous injection in having minimal off-target vector sequestration and reduced necessity for endothelial passage. Moreover, different AAV serotypes utilize distinct primary receptors for cell attachment and entry. AAV6 primarily binds to sialic acid and heparan, while AAV9 primarily binds to terminal galactose and uses the laminin receptor.[29,76] These

differences in vector sequestration, endothelial passage necessity, and receptor affinity likely together contribute to the different performance of AAVs in different delivery methods. We therefore expect more efficient AAV optimization using screening methods tailored to local vector application, and we expect that variant libraries based on AAV6 combined with these methods will yield further improved variants.

In the context of intramyocardial injections, in contrast to systemic applications, we propose *in vitro* and *ex vivo* assays we used may serve as predictors for vector performance, thereby providing useful platforms for optimization studies and validation in human tissue. The *in vitro* and *ex vivo* environments are lacking in complexity due to the absence of blood vessels and a competent immune system, allowing direct viral-to-cell contacts without competing vector absorption in off-target organs. These characteristics resemble the conditions of direct intramyocardial injection, where off-target transduction and contact with cellular and humoral immunity are minimized, and viral vectors do not need to pass the endothelial barrier.[41,77] Our results obtained with NRVMs closely predicted the outcomes of the mouse experiments. Additionally, comparison of natural AAV serotypes in various *in vitro* and *ex vivo* models has shown results largely consistent with those from *in vivo* experiments utilizing intramyocardial delivery,[29,62,68,78] further indicating high predictive power of these *in vitro* and *ex vivo* models.

Two limitations of this study should be acknowledged. First, our *in vivo* experiments were conducted up to 4 weeks post-injection, which may be insufficient to detect delayed changes in transgene expression or late-onset toxicity. However, AAV-mediated transgene expression is generally considered stable beyond 4 weeks post-injection,[24,25,79] making significant changes unlikely at later stages. Second, despite our best efforts, slight variability in the injection site location within the mouse hearts existed due to technical challenges. To mitigate this, we consistently sampled the lower half of the heart, ensuring inclusion of the injection site and maintaining comparable tissue composition across samples.

Our study showed that AAV6 is more efficient in transducing cardiac tissue than AAV9 in the context of direct intramyocardial injection. Moreover, our *in vitro* assays of cardiomyocyte transduction represent a sensitive and selective method to predict *in vivo* vector performance in this setting, thus representing useful tools for future AAV vector engineering efforts. These insights will facilitate the development of locally applied cardiac gene therapies and set the stage for subsequent optimization efforts and translational studies.

MATERIALS AND METHODS

Animal experiments

All animal experiments were approved and monitored by the Animal Ethics Committees of the UMC Utrecht and Amsterdam UMC and conducted in accordance with Directive 2010/63/EU of the European Parliament and with the “Guide for the Care and Use of Laboratory Animals.”

Mouse experiments

Eight-week-old FVB female mice and C57BL/6J male mice were acquired from Janvier (Le Genest-Saint-Isle, France) and acclimated for a week before the operation. Mice were injected subcutaneously with buprenorphine (0.075 mg/kg) and carprofen (0.05 mg/kg) for analgesia, at least 30 min prior to surgery. Anesthesia was induced with 4% isoflurane in 1 L/min O_2. Mice were shaved, intubated, and placed on a heating mat to maintain body temperature. Subsequently, an analgesic mixture consisting of lidocaine (2 mg/kg) and bupivacaine (3 mg/kg) was applied subcutaneously at the site of the incision. Anesthesia was maintained using ventilation with 1.5% isoflurane in 1 L/min O_2 mixed with 0.15 L/min air. Left thoracotomy was performed to expose the apex of the heart at the fourth intercostal space. To inject the AAV vectors into the apex, a 10 μL Hamilton syringe (RN1701) fitted with a 31-G needle (13 mm, point style 4) was inserted from the anterior left ventricle toward the apex in a shallow angle to prevent penetrating into the lumen. The viral vector solution was slowly administered in four injections of 5 μL at the same location to administer a total of 20 μL of viral vector (5×10^{12} or 5×10^{13} vg/mL). The thoracotomy and skin were closed with a C-1 12-mm cutting needle with a 6-0 silicone-coated braided silk wire. Post-surgical analgesia consisted of four days of *ad libitum* carprofen (0.06 mg/mL) in drinking water and high caloric wet food.

Pig experiments

Four male Topigs Norsvin pigs (Van Beek SPF Varkens; weight 34.13 [6.67] kg) were conventionally housed in stables with hay bedding, day/night light cycles of 12 h, and fed with standard chow diet and water *ad libitum*. Three days before AAV injection, we initiated an oral immunosuppressive regimen consisting of cyclosporine A (100 mg/kg/day) and prednisolone (2 mg/kg/day) to avert an immune response against the viral vectors. One day before AAV injection, anticoagulation treatment was initiated (for consistency with other gene therapy studies in our laboratory) with carbasalate calcium (320 mg one day before surgery and 160 mg on the day of the surgery), and analgesia was achieved with a buprenorphine patch (20 μg/h). Both the immunosuppressive and the anticoagulation therapies were maintained until termination. On the day of the injection, animals were premedicated intramuscularly with ketamine (10 mg/kg), midazolam (0.4 mg/kg), and atropine (0.05 mg/kg). Anesthesia induction was achieved with thiopental (4 mg/kg), followed by endotracheal intubation and anesthesia maintenance with a constant-rate infusion of midazolam (0.4 mg/kg/h), sufentanil (2.5 μg/kg/h), and *cis*-atracurium (0.7 mg/kg/h). Pigs were mechanically ventilated with a positive pressure ventilator with FiO_2 0.5, 1.5 L/min and an average frequency of 14 respirations/min under continuous capnography. Immunosuppressive treatment could not be assured orally during surgery; thus we performed a 2-h infusion of 200 mg in 100 mL of cyclosporine starting 1 hour before the injections. Pre-incisional analgesia was achieved with lidocaine (10 mg/mL), continued with a sternotomy and a full pericardiotomy to gain sub-epicardial access. Sternal homeostasis was assured using bone wax. Next, the heart was stabilized using a cardiac positioner (Starfish) ensuring hemodynamic stability, and three sutures (6-0

Prolene) were sewn to demarcate injection sites. We conducted four sub-epicardial injections using insulin syringes (BD Micro-Fine+), two of which contained 200 μL (high dose) of viral particle solution and two 20 μL (low dose) of viral particles solution (5×10^{12} vg/mL), the volume of each being dispensed in 40 s. Later, two drains were inserted in the pleural and pericardial cavities, anchored with a purse-string suture (2-0 FS-1 Vicryl) followed by the closure of the pericardium (6-0 C-1 Prolene) and the thorax (5 CCS Stainless steel). Additional analgesia was warranted using bupivacaine (2.5 mg/mL) in the incision after suturing the intramuscular planes (2 CTX plus and 0 MH plus, Vicryl) and prior to suturing the subcutaneous one (2-0 Vicryl); a continuous transdermal suture was performed to close the skin (3-0 PS-1 Monocryl). The chest drains were removed once exudate production was resolved.

Vector production

Capsid-pseudotyped AAV vectors with AAV2 ITRs containing *GFP* transgene driven by cTnT promoter were produced in 293T cells as previously reported.[40] AAV vectors were purified by using iodixanol density gradients and buffer exchanged to PBS + 0.001% pluronic using Amicon Ultra 15 mL centrifugal filters (100 kDa; Merck Life Science). Genomic titer was determined by qPCR using double-stranded plasmid DNA templates as standard curves with Fw: CAAAATTTGTGAAAGATTGACTGG; Rv: AAAGCCATACGGG AAGCAAT. AAV vectors in which *GFP* expression was driven by CMV promoter were provided by Revvity Gene Delivery.

In vitro transduction

NRVMs were isolated from 1- to 2-day-old rats as described previously.[80] NRVMs were seeded at a density of 4.5×10^5 cells per well in 24-well culture plates coated with a mixture of 12.5% fibronectin and 0.1% gelatin and kept in Tung medium (M199 supplemented with 200 U/L penicillin, 200 μg/L streptomycin, 20 μg/L vitamin B12, 1% MEM non-essential amino acids, 1% HEPES, 3.5 g/L glucose, and 2 mM L-glutamine) with 10% heat-inactivated fetal bovine serum (FBS). The purity of these NRVM preparations is around 74%. The medium was refreshed 1 day after plating and changed to Tung medium with 2% heat-inactivated FBS. On the same day, NRVMs were transduced with AAVs at an MOI of 10^4 vector genomes (vg)/cell for all groups except AAV9_10×, where an MOI of 10^5 vg/cell was applied.

Human iPSC-CMs were differentiated as previously reported,[81] seeded in culture plates coated with Matrigel and kept in BPEL medium.[82] The purity of these hiPSC-CM populations is around 86%. On the same day, cells were transduced with AAVs at an MOI of 5×10^4vg/cell. Medium was refreshed 1 day after transduction and refreshed every 2 days till analyses.

LAASs

Viable LAASs were obtained from patients with atrial fibrillation undergoing thoracoscopic ablation and included in the MARK AF registry (NL5006901819). The left atrial appendage was excised as standard of care during the procedure using a stapling device (Endo Gia stapler, Tyco Healthcare). All patients provided written informed consent.[83] Samples were immediately immersed in ice-cold, heparinized (2 mL/L) slicing solution containing 140 mM NaCl, 1.8 mM $CaCl_2 \cdot H_2O$, 4.7 mM KCl, 1 mM $MgCl_2 \cdot 6H_2O$, 10 mM glucose, 10 mM HEPES, and 30 mM 2,3-butanedione monoxime (pH 7.4, adjusted with NaOH). The tissue was embedded in 4% low melting point agarose (16520-100, Thermo Fisher Scientific) and mounted on a specimen holder.

LAASs were prepared using a vibrating microtome (7000 smz-2, Campden Instruments).[84] In a 4°C slicing solution bath, 380-μm slices were cut at 0.02 mm/s with pre-calibrated blades. The slices were then placed in a 37°C recovery solution with the same composition as the slicing solution but without 2,3-butanedione monoxime. Slices were allowed to recover for at least 1 hour before interventions were undertaken.

Prepared human LAASs were cultured in 6-well culture plates with culture inserts (PCHT06H48, Merck Life Science) in M199 medium (11150059, Thermo Fisher Scientific) supplemented with 3% penicillin/streptomycin and 1% ITS supplement (I3146, Merck Life Science). For transduction, 10^{11} vg of each AAV vector in 50 μL formulation buffer was added onto each slice (corresponding to an MOI of $\sim 2 \times 10^5$ vg/cell). One day post-transduction, LAASs were washed twice with PBS containing 3% penicillin/streptomycin and cultured in M199 medium supplemented with 1% penicillin/streptomycin and 1% ITS supplement. Medium was then refreshed daily until analyses were conducted.

RNA isolation, DNA isolation, cDNA synthesis, and RT-qPCR

Cells were collected in lysis buffer RA1 (Macherey-Nagel). Tissue samples were snap-frozen and preserved at −80°C, followed by homogenization with a SamplePrep CG-200-230 Freezer/Mill Compact Cryogenic Grinder (part #6775, Cole Parmer, Vernon Hills, IL) or kept in RNA preservation buffer immediately after imaging and homogenized using a T10 Basic Ultra Turrax homogenizer (0003737000, IKA-Werke) in lysis buffer RA1. Total RNA and DNA were isolated using NucleoSpin RNA mini kit (Macherey-Nagel) supplemented by DNA/RNA buffer set according to the manufacturer's protocol. Complementary DNA was transcribed from 200 to 1,000 ng of total RNA with oligo-dT primers (125 μM) and the Superscript II system (Thermo Fisher Scientific). qPCR was performed using the LightCycler 480 Real-Time PCR system (Roche Diagnostics). Relative mRNA expression of the transgene was normalized to that of the reference gene HPRT1 (mouse and human) or ACTB (pig). The number of vg was normalized to the number of diploid target cell genomes based on input DNA assuming 6.5 ng DNA per diploid genome.

Direct fluorescence microscopy

NRVMs

GFP fluorescence and bright field images were taken 3 days post-transduction using an inverted fluorescent microscope (ECLIPSE Ts2R, Nikon Europe, Amstelveen, the Netherlands). Identical exposure and magnification settings were applied for all wells. For

hiPSC-CMs, GFP fluorescence images were taken 5 days post-transduction using the same microscope, but images were captured with an external camera with fixed settings.

Mouse tissue

Mice were killed 4 weeks post-AAV injection. GFP fluorescence and bright field images were taken using a fluorescence stereo microscope (M205 FCA, Leica Microsystems) shortly after the heart was isolated. Identical capturing and analysis settings were applied for all animals.

Immunofluorescence staining

Mouse tissue

Tissues were fixed overnight in 4% paraformaldehyde solution in PBS, transferred to 70% ethanol, embedded in Paraplast (Leica Biosystems) and sectioned at 7 μm. Sections were deparaffinized in xylene and rehydrated by an ethanol series of descending concentrations. For antigen retrieval, sections were boiled in unmasking solution (H3300, VectorLabs). Sections were treated with 0.1% Triton X-100 in PBS for 30 min, and non-specific binding sites were blocked using PBS containing 4% bovine serum albumin and incubated overnight with anti-GFP (1:500; ab13970, Abcam), anti-Tnnt2 (1:100; MA5-12960, Thermo Fisher Scientific), and anti-dystrophin (1:150; ab15277, Abcam) antibodies. The next day, the sections were incubated with secondary antibodies conjugated with Alexa Fluor 488, 555, and 647 in combination with the nucleic acid stain DAPI (1 μg/mL; D9542, Merck Life Science). Sections were then mounted, and fluorescence images were acquired using a DM6000 fluorescence microscope (Leica Microsystems).

Pig tissue

Cardiac tissue was snap-frozen and subsequently processed as described above for mouse tissue. Sections were incubated overnight with anti-GFP (1:500; ab13970, Abcam) and anti-Tnnt2 (1:100; MA5-12960, Thermo Fisher) antibodies. The next day, the sections were incubated with secondary antibodies conjugated with Alexa Fluor 488 and 647 in combination with Alexa Fluor 555-conjugated wheat germ agglutinin (1:250, W32464, Thermo Fisher Scientific) and DAPI (1 μg/mL, D9542, Sigma) for visualization of the plasma membrane and cell nucleus, respectively. Sections were then mounted, and fluorescence images were acquired using the aforementioned Leica DM6000 fluorescence microscope.

Picrosirius red staining

Sections were deparaffinized in xylene, rehydrated by an ethanol series of descending concentration, and stained with picrosirius red (P6744, Sigma-Aldrich) to visualize collagen. Stained sections were imaged using a DM5000 fluorescence microscope (Leica Microsystems). Image analysis was performed on 10 non-contiguous sections from the apex of the heart using ImageJ.

Statistical analysis

Statistical analysis was performed using Student's t test or analysis of variance for repeated measurements (ANOVA) with *post-hoc* Fisher's least significant difference (LSD) test in Prism 10 (GraphPad Software, Boston, MA).

DATA AVAILABILITY

All data generated in this study are included in this published article and in the supplemental information. All these data will be made available upon reasonable request.

ACKNOWLEDGMENTS

This work was supported by European Research Council (starting grant 714866, proof-of-concept grant 899422 and 101081921 to G.J.J.B., proof-of-concept grant 101138069 to J.P.G.S.), Health Holland (LentiPace II to G.J.J.B. and H.L.T.), Horizon 2020 (Eurostars E114245 and E115484 to G.J.J.B. and V.M.C., project TECHNOBEAT, grant number 66724 and RIA-HEAL, grant number 101056712 to J.P.G.S.), Dutch Research Council (Open Technology Program 18485 to H.L.T. and G.J.J.B.; OCENW.GROOT.2019.029 to V.M.C., 2021/TTW/01038252 to G.J.J.B. and J.P.G.S.), European Innovation Council (Pathfinder Challenges Nav1.5-CARED to V.M.C. and G.J.J.B., TRANSITION Project 101099608 TRACTION to V.M.C. and G.J.J.B.), H2020 Marie Sklodowska-Curie Actions COFUND program (No 801540 to J.P.G.S.), and RegMed XB (Cardiovascular Moonshot to A.A.F.d.V.).

AUTHOR CONTRIBUTIONS

Conceptualization, J.W., T.J., V.M.C., and G.J.J.B.; formal analysis, J.W. and T.J.; funding acquisition, G.J.J.B., H.L.T., O.F.K., V.M.C., J.P.G.S., and A.A.F.d.V.; investigation, J.W., T.J., A.C.-B., Z.D., R.N.V., E.E.B., A.R.B., M.K., Y.Y., J.M.V., M.S.J., and T.C.G.; resources, S.S., C.T., C.I.B., A.A.F.d.V.; supervision, G.J.J.B., V.M.C., J.P.G.S., and S.C.A. d.J.; visualization, J.W., T.J., and A.C.-B.; writing—original draft, J.W., T.J., and A. C.-B.; writing—review & editing, all authors.

DECLARATION OF INTERESTS

O.F.K., H.L.T., and G.J.J.B. are co-founders of PacingCure BV and report ownership interest in PacingCure BV. J.W. and E.E.B. are employees of PacingCure BV. T.J., V.M.C., and G.J.J.B. have pending patent applications related to this work.

SUPPLEMENTAL INFORMATION

Supplemental information can be found online at https://doi.org/10.1016/j.omtm.2025.101532.

REFERENCES

1. Roth, G.A., Mensah, G.A., Johnson, C.O., Addolorato, G., Ammirati, E., Baddour, L. M., Barengo, N.C., Beaton, A.Z., Benjamin, E.J., Benziger, C.P., et al. (2020). Global Burden of Cardiovascular Diseases and Risk Factors, 1990-2019: Update From the GBD 2019 Study. J. Am. Coll. Cardiol. *76*, 2982–3021. https://doi.org/10.1016/j.jacc.2020.11.010.
2. Bulcha, J.T., Wang, Y., Ma, H., Tai, P.W.L., and Gao, G. (2021). Viral vector platforms within the gene therapy landscape. Signal Transduct. Target. Ther. *6*, 53. https://doi.org/10.1038/s41392-021-00487-6.
3. Konkalmatt, P.R., Beyers, R.J., O'Connor, D.M., Xu, Y., Seaman, M.E., and French, B.A. (2013). Cardiac-selective expression of extracellular superoxide dismutase after systemic injection of adeno-associated virus 9 protects the heart against post-myocardial infarction left ventricular remodeling. Circ. Cardiovasc. Imaging *6*, 478–486. https://doi.org/10.1161/CIRCIMAGING.112.000320.
4. Singh, M., Brooks, A., Toofan, P., and McLuckie, K. (2024). Selection of appropriate non-clinical animal models to ensure translatability of novel AAV-gene therapies to the clinic. Gene Ther. *31*, 56–63. https://doi.org/10.1038/s41434-023-00417-x.
5. Philippidis, A. (2024). Patient Dies in Beam Trial of Sickle Cell Disease Candidate; Company Cites Conditioning. Hum. Gene Ther. *35*, 951–954. https://doi.org/10.1089/hum.2024.111924.
6. Miguel-Dos-Santos, R., and Cingolani, E. (2023). The hunt for novel AAV capsids with improved cardiac tropism. Mol. Ther. Methods Clin. Dev. *31*, 101124. https://doi.org/10.1016/j.omtm.2023.101124.
7. Kim, Y., Landstrom, A.P., Shah, S.H., Wu, J.C., and Seidman, C.E.; American Heart Association (2024). Gene Therapy in Cardiovascular Disease: Recent Advances and Future Directions in Science: A Science Advisory From the

American Heart Association. Circulation *150*, e471–e480. https://doi.org/10.1161/CIR.0000000000001296.

8. Greenberg, B., Butler, J., Felker, G.M., Ponikowski, P., Voors, A.A., Desai, A.S., Barnard, D., Bouchard, A., Jaski, B., Lyon, A.R., et al. (2016). Calcium upregulation by percutaneous administration of gene therapy in patients with cardiac disease (CUPID 2): a randomised, multinational, double-blind, placebo-controlled, phase 2b trial. Lancet *387*, 1178–1186. https://doi.org/10.1016/S0140-6736(16)00082-9.

9. Lyon, A.R., Babalis, D., Morley-Smith, A.C., Hedger, M., Suarez Barrientos, A., Foldes, G., Couch, L.S., Chowdhury, R.A., Tzortzis, K.N., Peters, N.S., et al. (2020). Investigation of the safety and feasibility of AAV1/SERCA2a gene transfer in patients with chronic heart failure supported with a left ventricular assist device - the SERCA-LVAD TRIAL. Gene Ther. *27*, 579–590. https://doi.org/10.1038/s41434-020-0171-7.

10. Study Assessing Left Ventricular Administration of a Genetic Medicine Directing Organ Regeneration in Heart Failure (SALVADOR-HF). (2025). https://clinicaltrials.gov/study/NCT06831825.

11. Myocardial Telomere Recapping Study for Dilated Cardiomyopathy (MERCURY-DCM). (2023). https://clinicaltrials.gov/study/NCT05837143.

12. Multi-center, Open-label, Single-ascending Dose Study of Safety and Tolerability of TN-201 in Adults With Symptomatic MYBPC3 Mutation-associated HCM (MyPEAK-1). (2023). https://clinicaltrials.gov/study/NCT05836259.

13. Open-label, Dose Escalation Study of Safety and Preliminary Efficacy of TN-401 in Adults With PKP2 Mutation-associated ARVC (RIDGE-1). (2025). https://clinicaltrials.gov/study/NCT06228924.

14. Non-interventional Study of Seroprevalence of Pre-existing Antibodies Against Adenovirus-associated Virus Vector (AAV9) and the Progression of Disease in Patients With Plakophilin 2 (PKP2)-Associated Arrhythmogenic Right Ventricular Cardiomyopathy (ARVC) (RIDGE). (2024). https://clinicaltrials.gov/study/NCT06311708.

15. A Phase 1, Dose Escalation Trial of RP-A601 in Subjects With PKP2 Variant-Mediated Arrhythmogenic Cardiomyopathy (PKP2-ACM). (2024). https://clinicaltrials.gov/study/NCT05885412.

16. Pupo, A., Fernández, A., Low, S.H., François, A., Suárez-Amarán, L., and Samulski, R.J. (2022). AAV vectors: The Rubik's cube of human gene therapy. Mol. Ther. *30*, 3515–3541. https://doi.org/10.1016/j.ymthe.2022.09.015.

17. Inagaki, K., Fuess, S., Storm, T.A., Gibson, G.A., McTiernan, C.F., Kay, M.A., and Nakai, H. (2006). Robust systemic transduction with AAV9 vectors in mice: efficient global cardiac gene transfer superior to that of AAV8. Mol. Ther. *14*, 45–53. https://doi.org/10.1016/j.ymthe.2006.03.014.

18. Kupatt, C., Hinkel, R., Pfosser, A., El-Aouni, C., Wuchrer, A., Fritz, A., Globisch, F., Thormann, M., Horstkotte, J., Lebherz, C., et al. (2010). Cotransfection of vascular endothelial growth factor-A and platelet-derived growth factor-B via recombinant adeno-associated virus resolves chronic ischemic malperfusion role of vessel maturation. J. Am. Coll. Cardiol. *56*, 414–422. https://doi.org/10.1016/j.jacc.2010.03.050.

19. Byrne, M.J., Power, J.M., Preovolos, A., Mariani, J.A., Hajjar, R.J., and Kaye, D.M. (2008). Recirculating cardiac delivery of AAV2/1SERCA2a improves myocardial function in an experimental model of heart failure in large animals. Gene Ther. *15*, 1550–1557. https://doi.org/10.1038/gt.2008.120.

20. Katz, M.G., Fargnoli, A.S., Williams, R.D., Steuerwald, N.M., Isidro, A., Ivanina, A. V., Sokolova, I.M., and Bridges, C.R. (2014). Safety and efficacy of high-dose adeno-associated virus 9 encoding sarcoplasmic reticulum Ca(2+) adenosine triphosphatase delivered by molecular cardiac surgery with recirculating delivery in ovine ischemic cardiomyopathy. J. Thorac. Cardiovasc. Surg. *148*, 1065–discussion1072. 1073e1-2; discussion 1072-1063. https://doi.org/10.1016/j.jtcvs.2014.05.070.

21. Beeri, R., Chaput, M., Guerrero, J.L., Kawase, Y., Yosefy, C., Abedat, S., Karakikes, I., Morel, C., Tisosky, A., Sullivan, S., et al. (2010). Gene delivery of sarcoplasmic reticulum calcium ATPase inhibits ventricular remodeling in ischemic mitral regurgitation. Circ. Heart Fail. *3*, 627–634. https://doi.org/10.1161/CIRCHEARTFAILURE.109.891184.

22. Ai, J., Li, J., Gessler, D.J., Su, Q., Wei, Q., Li, H., and Gao, G. (2017). Adeno-associated virus serotype rh.10 displays strong muscle tropism following intraperitoneal delivery. Sci. Rep. *7*, 40336. https://doi.org/10.1038/srep40336.

23. Pozsgai, E.R., Griffin, D.A., Heller, K.N., Mendell, J.R., and Rodino-Klapac, L.R. (2017). Systemic AAV-Mediated beta-Sarcoglycan Delivery Targeting Cardiac and Skeletal Muscle Ameliorates Histological and Functional Deficits in LGMD2E Mice. Mol. Ther. *25*, 855–869. https://doi.org/10.1016/j.ymthe.2017.02.013.

24. Zincarelli, C., Soltys, S., Rengo, G., and Rabinowitz, J.E. (2008). Analysis of AAV serotypes 1-9 mediated gene expression and tropism in mice after systemic injection. Mol. Ther. *16*, 1073–1080. https://doi.org/10.1038/mt.2008.76.

25. Zincarelli, C., Soltys, S., Rengo, G., Koch, W.J., and Rabinowitz, J.E. (2010). Comparative cardiac gene delivery of adeno-associated virus serotypes 1-9 reveals that AAV6 mediates the most efficient transduction in mouse heart. Clin. Transl. Sci. *3*, 81–89. https://doi.org/10.1111/j.1752-8062.2010.00190.x.

26. Tabebordbar, M., Lagerborg, K.A., Stanton, A., King, E.M., Ye, S., Tellez, L., Krunnfusz, A., Tavakoli, S., Widrick, J.J., Messemer, K.A., et al. (2021). Directed evolution of a family of AAV capsid variants enabling potent muscle-directed gene delivery across species. Cell *184*, 4919–4938.e22. https://doi.org/10.1016/j.cell.2021.08.028.

27. Walkey, C.J., Snow, K.J., Bulcha, J., Cox, A.R., Martinez, A.E., Ljungberg, M.C., Lanza, D.G., De Giorgi, M., Chuecos, M.A., Alves-Bezerra, M., et al. (2025). A comprehensive atlas of AAV tropism in the mouse. Mol. Ther. *33*, 1282–1299. https://doi.org/10.1016/j.ymthe.2025.01.041.

28. Cheng, Z., Easter, E., Rollosson, L.M., Feathers, C., Leong, J., Lim, B., Jones, S., Woods, J., Parvathaneni, A., Reid, C.A., et al. (2023). Engineering Novel AAV Capsids for Cardiac Gene Delivery. Mol. Ther. *31*, 1–794. https://doi.org/10.1016/j.ymthe.2023.04.017.

29. Ambrosi, C.M., Sadananda, G., Han, J.L., and Entcheva, E. (2019). Adeno-Associated Virus Mediated Gene Delivery: Implications for Scalable in vitro and in vivo Cardiac Optogenetic Models. Front. Physiol. *10*, 168. https://doi.org/10.3389/fphys.2019.00168.

30. Kok, C.Y., Tsurusaki, S., Cabanes-Creus, M., Igoor, S., Rao, R., Skelton, R., Liao, S.H. Y., Ginn, S.L., Knight, M., Scott, S., et al. (2023). Development of new adeno-associated virus capsid variants for targeted gene delivery to human cardiomyocytes. Mol. Ther. Methods Clin. Dev. *30*, 459–473. https://doi.org/10.1016/j.omtm.2023.08.010.

31. Fang, H., Lai, N.C., Gao, M.H., Miyanohara, A., Roth, D.M., Tang, T., and Hammond, H.K. (2012). Comparison of adeno-associated virus serotypes and delivery methods for cardiac gene transfer. Hum. Gene Ther. Methods *23*, 234–241. https://doi.org/10.1089/hgtb.2012.105.

32. Weber-Adrian, D., Kofoed, R.H., Silburt, J., Noroozian, Z., Shah, K., Burgess, A., Rideout, S., Kügler, S., Hynynen, K., and Aubert, I. (2021). Systemic AAV6-synapsin-GFP administration results in lower liver biodistribution, compared to AAV1&2 and AAV9, with neuronal expression following ultrasound-mediated brain delivery. Sci. Rep. *11*, 1934. https://doi.org/10.1038/s41598-021-81046-5.

33. Piras, B.A., Tian, Y., Xu, Y., Thomas, N.A., O'Connor, D.M., and French, B.A. (2016). Systemic injection of AAV9 carrying a periostin promoter targets gene expression to a myofibroblast-like lineage in mouse hearts after reperfused myocardial infarction. Gene Ther. *23*, 469–478. https://doi.org/10.1038/gt.2016.20.

34. Boink, G.J.J., Christoffels, V.M., Robinson, R.B., and Tan, H.L. (2015). The past, present, and future of pacemaker therapies. Trends Cardiovasc. Med. *25*, 661–673. https://doi.org/10.1016/j.tcm.2015.02.005.

35. Gabisonia, K., Prosdocimo, G., Aquaro, G.D., Carlucci, L., Zentilin, L., Secco, I., Ali, H., Braga, L., Gorgodze, N., Bernini, F., et al. (2019). MicroRNA therapy stimulates uncontrolled cardiac repair after myocardial infarction in pigs. Nature *569*, 418–422. https://doi.org/10.1038/s41586-019-1191-6.

36. Burjanadze, G., Gorgodze, N., Aquaro, G.D., Gabisonia, K., Carlucci, L., Pachauri, M., Turreni, F., Secco, I., Bernini, F., Zentilin, L., et al. (2025). Delayed miR-199a Administration After Myocardial Infarction Precludes Pro-Regenerative Effects. JACC. Basic Transl. Sci. *10*, 634–649. https://doi.org/10.1016/j.jacbts.2024.12.014.

37. Bouwman, M., de Bakker, D.E.M., Honkoop, H., Giovou, A.E., Versteeg, D., Boender, A.R., Nguyen, P.D., Slotboom, M., Colquhoun, D., Vigil-Garcia, M., et al. (2025). Cross-species comparison reveals that Hmga1 reduces H3K27me3 levels to promote cardiomyocyte proliferation and cardiac regeneration. Nat. Cardiovasc. Res. *4*, 64–82. https://doi.org/10.1038/s44161-024-00588-9.

38. Liu, S., Li, K., Wagner Florencio, L., Tang, L., Heallen, T.R., Leach, J.P., Wang, Y., Grisanti, F., Willerson, J.T., Perin, E.C., et al. (2021). Gene therapy knockdown of Hippo signaling induces cardiomyocyte renewal in pigs after myocardial infarction. Sci. Transl. Med. *13*, eabd6892. https://doi.org/10.1126/scitranslmed.abd6892.

39. Mundisugih, J., Kumar, S., and Kizana, E. (2024). Adeno-associated virus-mediated gene therapy for cardiac tachyarrhythmia: A systematic review and meta-analysis. Heart Rhythm *21*, 939–949. https://doi.org/10.1016/j.hrthm.2024.02.001.

40. Wang, J., Verkerk, A.O., Wilders, R., Zhang, Y., Zhang, K., Prakosa, A., Rivaud, M. R., Marsman, E.M.J., Boender, A.R., Klerk, M., et al. (2025). SCN10A-short gene therapy to restore conduction and protect against malignant cardiac arrhythmias. Eur. Heart J. *46*, 1747–1762. https://doi.org/10.1093/eurheartj/ehaf053.

41. Ishikawa, K., Tilemann, L., Fish, K., and Hajjar, R.J. (2011). Gene delivery methods in cardiac gene therapy. J. Gene Med. *13*, 566–572. https://doi.org/10.1002/jgm.1609.

42. Schneider, C., Jaquet, K., Malisius, R., Geidel, S., Bahlmann, E., Boczor, S., Rau, T., Antz, M., Kuck, K.H., and Krause, K. (2007). Attenuation of cardiac remodelling by endocardial injection of erythropoietin: ultrasonic strain-rate imaging in a model of hibernating myocardium. Eur. Heart J. *28*, 499–509. https://doi.org/10.1093/eurheartj/ehl439.

43. Lau, D.H., Clausen, C., Sosunov, E.A., Shlapakova, I.N., Anyukhovsky, E.P., Danilo, P., Jr., Rosen, T.S., Kelly, C., Duffy, H.S., Szabolcs, M.J., et al. (2009). Epicardial border zone overexpression of skeletal muscle sodium channel SkM1 normalizes activation, preserves conduction, and suppresses ventricular arrhythmia: an in silico, in vivo, in vitro study. Circulation *119*, 19–27. https://doi.org/10.1161/CIRCULATIONAHA.108.809301.

44. Bish, L.T., Sleeper, M.M., Brainard, B., Cole, S., Russell, N., Withnall, E., Arndt, J., Reynolds, C., Davison, E., Sanmiguel, J., et al. (2008). Percutaneous transendocardial delivery of self-complementary adeno-associated virus 6 achieves global cardiac gene transfer in canines. Mol. Ther. *16*, 1953–1959. https://doi.org/10.1038/mt.2008.202.

45. Gao, G., Bish, L.T., Sleeper, M.M., Mu, X., Sun, L., Lou, Y., Duan, J., Hu, C., Wang, L., and Sweeney, H.L. (2011). Transendocardial delivery of AAV6 results in highly efficient and global cardiac gene transfer in rhesus macaques. Hum. Gene Ther. *22*, 979–984. https://doi.org/10.1089/hum.2011.042.

46. Prasad, K.M.R., Smith, R.S., Xu, Y., and French, B.A. (2011). A single direct injection into the left ventricular wall of an adeno-associated virus 9 (AAV9) vector expressing extracellular superoxide dismutase from the cardiac troponin-T promoter protects mice against myocardial infarction. J. Gene Med. *13*, 333–341. https://doi.org/10.1002/jgm.1576.

47. Palomeque, J., Chemaly, E.R., Colosi, P., Wellman, J.A., Zhou, S., Del Monte, F., and Hajjar, R.J. (2007). Efficiency of eight different AAV serotypes in transducing rat myocardium in vivo. Gene Ther. *14*, 989–997. https://doi.org/10.1038/sj.gt.3302895.

48. Merentie, M., Lottonen-Raikaslehto, L., Parviainen, V., Huusko, J., Pikkarainen, S., Mendel, M., Laham-Karam, N., Kärjä, V., Rissanen, R., Hedman, M., and Ylä-Herttuala, S. (2016). Efficacy and safety of myocardial gene transfer of adenovirus, adeno-associated virus and lentivirus vectors in the mouse heart. Gene Ther. *23*, 296–305. https://doi.org/10.1038/gt.2015.114.

49. Huusko, J., Lottonen, L., Merentie, M., Gurzeler, E., Anisimov, A., Miyanohara, A., Alitalo, K., Tavi, P., and Ylä-Herttuala, S. (2012). AAV9-mediated VEGF-B gene transfer improves systolic function in progressive left ventricular hypertrophy. Mol. Ther. *20*, 2212–2221. https://doi.org/10.1038/mt.2012.145.

50. Moulay, G., Ohtani, T., Ogut, O., Guenzel, A., Behfar, A., Zakeri, R., Haines, P., Storlie, J., Bowen, L., Pham, L., et al. (2015). Cardiac AAV9 Gene Delivery Strategies in Adult Canines: Assessment by Long-term Serial SPECT Imaging of Sodium Iodide Symporter Expression. Mol. Ther. *23*, 1211–1221. https://doi.org/10.1038/mt.2015.78.

51. Fish, K.M., Ladage, D., Kawase, Y., Karakikes, I., Jeong, D., Ly, H., Ishikawa, K., Hadri, L., Tilemann, L., Muller-Ehmsen, J., et al. (2013). AAV9.I-1c delivered via direct coronary infusion in a porcine model of heart failure improves contractility and mitigates adverse remodeling. Circ. Heart Fail. *6*, 310–317. https://doi.org/10.1161/CIRCHEARTFAILURE.112.971325.

52. Li, J., Kelly, S.C., Ivey, J.R., Thorne, P.K., Yamada, K.P., Aikawa, T., Mazurek, R., Turk, J.R., Silva, K.A.S., Amin, A.R., et al. (2022). Distribution of cardiomyocyte-selective adeno-associated virus serotype 9 vectors in swine following intracoronary and intravenous infusion. Physiol. Genomics *54*, 261–272. https://doi.org/10.1152/physiolgenomics.00032.2022.

53. Pleger, S.T., Shan, C., Ksienzyk, J., Bekeredjian, R., Boekstegers, P., Hinkel, R., Schinkel, S., Leuchs, B., Ludwig, J., Qiu, G., et al. (2011). Cardiac AAV9-S100A1 gene therapy rescues post-ischemic heart failure in a preclinical large animal model. Sci. Transl. Med. *3*, 92ra64. https://doi.org/10.1126/scitranslmed.3002097.

54. Ji, J., Lefebvre, E., and Laporte, J. (2024). Comparative in vivo characterization of newly discovered myotropic adeno-associated vectors. Skelet. Muscle *14*, 9. https://doi.org/10.1186/s13395-024-00341-7.

55. Paneda, A., Vanrell, L., Mauleon, I., Crettaz, J.S., Berraondo, P., Timmermans, E.J., Beattie, S.G., Twisk, J., van Deventer, S., Prieto, J., et al. (2009). Effect of adeno-associated virus serotype and genomic structure on liver transduction and biodistribution in mice of both genders. Hum. Gene Ther. *20*, 908–917. https://doi.org/10.1089/hum.2009.031.

56. Mathiesen, S.N., Lock, J.L., Schoderboeck, L., Abraham, W.C., and Hughes, S.M. (2020). CNS Transduction Benefits of AAV-PHP.eB over AAV9 Are Dependent on Administration Route and Mouse Strain. Mol. Ther. Methods Clin. Dev. *19*, 447–458. https://doi.org/10.1016/j.omtm.2020.10.011.

57. He, T., Itano, M.S., Earley, L.F., Hall, N.E., Riddick, N., Samulski, R.J., and Li, C. (2019). The Influence of Murine Genetic Background in Adeno-Associated Virus Transduction of the Mouse Brain. Hum. Gene Ther. Clin. Dev. *30*, 169–181. https://doi.org/10.1089/humc.2019.030.

58. Chuecos, M.A., and Lagor, W.R. (2024). Liver directed adeno-associated viral vectors to treat metabolic disease. J. Inherit. Metab. Dis. *47*, 22–40. https://doi.org/10.1002/jimd.12637.

59. Bohlen, M.O., McCown, T.J., Powell, S.K., El-Nahal, H.G., Daw, T., Basso, M.A., Sommer, M.A., and Samulski, R.J. (2020). Adeno-Associated Virus Capsid-Promoter Interactions in the Brain Translate from Rat to the Nonhuman Primate. Hum. Gene Ther. *31*, 1155–1168. https://doi.org/10.1089/hum.2020.196.

60. Gonzalez-Sandoval, A., Pekrun, K., Tsuji, S., Zhang, F., Hung, K.L., Chang, H.Y., and Kay, M.A. (2023). The AAV capsid can influence the epigenetic marking of rAAV delivered episomal genomes in a species dependent manner. Nat. Commun. *14*, 2448. https://doi.org/10.1038/s41467-023-38106-3.

61. Davidoff, A.M., Ng, C.Y.C., Zhou, J., Spence, Y., and Nathwani, A.C. (2003). Sex significantly influences transduction of murine liver by recombinant adeno-associated viral vectors through an androgen-dependent pathway. Blood *102*, 480–488. https://doi.org/10.1182/blood-2002-09-2889.

62. Rapti, K., Stillitano, F., Karakikes, I., Nonnenmacher, M., Weber, T., Hulot, J.S., and Hajjar, R.J. (2015). Effectiveness of gene delivery systems for pluripotent and differentiated cells. Mol. Ther. Methods Clin. Dev. *2*, 14067. https://doi.org/10.1038/mtm.2014.67.

63. Kikuchi, K., McDonald, A.D., Sasano, T., and Donahue, J.K. (2005). Targeted modification of atrial electrophysiology by homogeneous transmural atrial gene transfer. Circulation *111*, 264–270. https://doi.org/10.1161/01.CIR.0000153338.47507.83.

64. Nyns, E.C.A., Poelma, R.H., Volkers, L., Plomp, J.J., Bart, C.I., Kip, A.M., van Brakel, T.J., Zeppenfeld, K., Schalij, M.J., Zhang, G.Q., et al. (2019). An automated hybrid bioelectronic system for autogenous restoration of sinus rhythm in atrial fibrillation. Sci. Transl. Med. *11*, eaau6447. https://doi.org/10.1126/scitranslmed.aau6447.

65. Mazurek, R., Tharakan, S., Mavropoulos, S.A., Singleton, D.T., Bikou, O., Sakata, T., Kariya, T., Yamada, K., Kohlbrenner, E., Liang, L., et al. (2024). AAV delivery strategy with mechanical support for safe and efficacious cardiac gene transfer in swine. Nat. Commun. *15*, 10450. https://doi.org/10.1038/s41467-024-54635-x.

66. Lampela, J., Pajula, J., Järveläinen, N., Siimes, S., Laham-Karam, N., Kivelä, A., Mushimiyimana, I., Nurro, J., Hartikainen, J., and Ylä-Herttuala, S. (2024). Caridac vein retroinjections provide an efficient approach for global left ventricular gene transfer with adenovirus and adeno-associated virus. Sci. Rep. *14*, 1467. https://doi.org/10.1038/s41598-024-51712-5.

67. Costa Verdera, H., Kuranda, K., and Mingozzi, F. (2020). AAV Vector Immunogenicity in Humans: A Long Journey to Successful Gene Transfer. Mol. Ther. *28*, 723–746. https://doi.org/10.1016/j.ymthe.2019.12.010.

68. Nunez-Toldra, R., Del Canizo, A., Secco, I., Nicastro, L., Giacca, M., and Terracciano, C.M. (2023). Living myocardial slices for the study of nucleic acid-based therapies. Front. Bioeng. Biotechnol. *11*, 1275945. https://doi.org/10.3389/fbioe.2023.1275945.

69. McTiernan, C.F., Mathier, M.A., Zhu, X., Xiao, X., Klein, E., Swan, C.H., Mehdi, H., Gibson, G., Trichel, A.M., Glorioso, J.C., et al. (2007). Myocarditis following adeno-associated viral gene expression of human soluble TNF receptor (TNFRII-Fc) in baboon hearts. Gene Ther. *14*, 1613–1622. https://doi.org/10.1038/sj.gt.3303020.

70. Mays, L.E., and Wilson, J.M. (2011). The complex and evolving story of T cell activation to AAV vector-encoded transgene products. Mol. Ther. *19*, 16–27. https://doi.org/10.1038/mt.2010.250.

71. Yang, L., Jiang, J., Drouin, L.M., Agbandje-McKenna, M., Chen, C., Qiao, C., Pu, D., Hu, X., Wang, D.Z., Li, J., and Xiao, X. (2009). A myocardium tropic adeno-associated virus (AAV) evolved by DNA shuffling and in vivo selection. Proc. Natl. Acad. Sci. USA *106*, 3946–3951. https://doi.org/10.1073/pnas.0813207106.

72. Rode, L., Bär, C., Groß, S., Rossi, A., Meumann, N., Viereck, J., Abbas, N., Xiao, K., Riedel, I., Gietz, A., et al. (2022). AAV capsid engineering identified two novel variants with improved in vivo tropism for cardiomyocytes. Mol. Ther. *30*, 3601–3618. https://doi.org/10.1016/j.ymthe.2022.07.003.

73. Pulicherla, N., Shen, S., Yadav, S., Debbink, K., Govindasamy, L., Agbandje-McKenna, M., and Asokan, A. (2011). Engineering liver-detargeted AAV9 vectors for cardiac and musculoskeletal gene transfer. Mol. Ther. *19*, 1070–1078. https://doi.org/10.1038/mt.2011.22.

74. Asokan, A., Conway, J.C., Phillips, J.L., Li, C., Hegge, J., Sinnott, R., Yadav, S., DiPrimio, N., Nam, H.J., Agbandje-McKenna, M., et al. (2010). Reengineering a receptor footprint of adeno-associated virus enables selective and systemic gene transfer to muscle. Nat. Biotechnol. *28*, 79–82. https://doi.org/10.1038/nbt.1599.

75. Ishikawa, K., Weber, T., and Hajjar, R.J. (2018). Human Cardiac Gene Therapy. Circ. Res. *123*, 601–613. https://doi.org/10.1161/CIRCRESAHA.118.311587.

76. Li, C., and Samulski, R.J. (2020). Engineering adeno-associated virus vectors for gene therapy. Nat. Rev. Genet. *21*, 255–272. https://doi.org/10.1038/s41576-019-0205-4.

77. Vekstein, A.M., Wendell, D.C., DeLuca, S., Yan, R., Chen, Y., Bishawi, M., Devlin, G. W., Asokan, A., Poss, K.D., Bowles, D.E., et al. (2022). Targeted Delivery for Cardiac Regeneration: Comparison of Intra-coronary Infusion and Intra-myocardial Injection in Porcine Hearts. Front. Cardiovasc. Med. *9*, 833335. https://doi.org/10.3389/fcvm.2022.833335.

78. Liu, Z., Klose, K., Neuber, S., Jiang, M., Gossen, M., and Stamm, C. (2020). Comparative analysis of adeno-associated virus serotypes for gene transfer in organotypic heart slices. J. Transl. Med. *18*, 437. https://doi.org/10.1186/s12967-020-02605-4.

79. Chen, Q., Zhai, H., Li, X., Ma, Y., Chen, B., Liu, F., Lai, H., Xie, J., He, C., Luo, J., et al. (2017). Recombinant adeno-associated virus serotype 9 in a mouse model of atherosclerosis: Determination of the optimal expression time in vivo. Mol. Med. Rep. *15*, 2090–2096. https://doi.org/10.3892/mmr.2017.6235.

80. Rohr, S., Schölly, D.M., and Kléber, A.G. (1991). Patterned growth of neonatal rat heart cells in culture. Morphological and electrophysiological characterization. Circ. Res. *68*, 114–130. https://doi.org/10.1161/01.res.68.1.114.

81. Maas, R.G., Lee, S., Harakalova, M., Blok, C.J.S., Goodyer, W.R., Hjortnaes, J., Doevendans, P.A., Van Laake, L.W., van der Velden, J., and Asselbergs, F.W. (2021). et al. Massive expansion and cryopreservation of functional human induced pluripotent stem cell-derived cardiomyocytes. STAR Protoc. *2*, 100334. https://doi.org/10.1016/j.xpro.2021.100334.

82. Ng, E.S., Davis, R., Stanley, E.G., and Elefanty, A.G. (2008). A protocol describing the use of a recombinant protein-based, animal product-free medium (APEL) for human embryonic stem cell differentiation as spin embryoid bodies. Nat. Protoc. *3*, 768–776. https://doi.org/10.1038/nprot.2008.42.

83. Krul, S.P.J., Driessen, A.H.G., van Boven, W.J., Linnenbank, A.C., Geuzebroek, G.S. C., Jackman, W.M., Wilde, A.A.M., de Bakker, J.M.T., and de Groot, J.R. (2011). Thoracoscopic video-assisted pulmonary vein antrum isolation, ganglionated plexus ablation, and periprocedural confirmation of ablation lesions: first results of a hybrid surgical-electrophysiological approach for atrial fibrillation. Circ. Arrhythm. Electrophysiol. *4*, 262–270. https://doi.org/10.1161/CIRCEP.111.961862.

84. Watson, S.A., Scigliano, M., Bardi, I., Ascione, R., Terracciano, C.M., and Perbellini, F. (2017). Preparation of viable adult ventricular myocardial slices from large and small mammals. Nat. Protoc. *12*, 2623–2639. https://doi.org/10.1038/nprot.2017.139.

OMTM, Volume *33*

Supplemental information

AAV6 vectors provide superior gene transfer compared to AAV9 vectors following intramyocardial administration

Jianan Wang (王嘉南), Timo Jonker, Aina Cervera-Barea, Zhenyu Dong, Ruud N. Visser, Evelien E. Birza, Arie R. Boender, Mischa Klerk, Yuting Yang (杨玉婷), Joyce Visser, Marlijn S. Jansen, Tom C. Grootswagers, Cindy I. Bart, Saskia C.A. de Jager, Silke Schrödel, Christian Thirion, Osne F. Kirzner, Hanno L. Tan, Antoine A.F. de Vries, Joost P.G. Sluijter, Klaus Neef, Joris R. de Groot, Vincent M. Christoffels, and Gerard J.J. Boink

Supplemental information

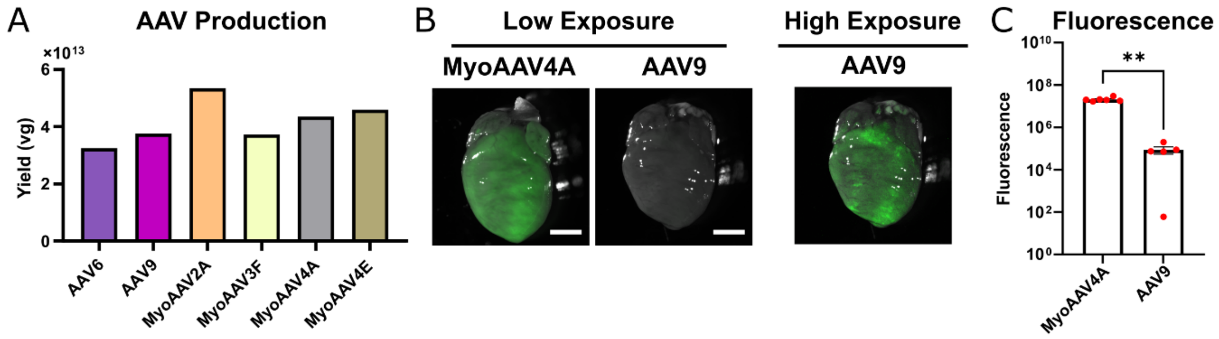

Figure S1. The production and validation of AAV vectors.

(**A**) Yields are similar among different AAV vector pseudotypes. (**B**) Typical heart images of female FVB mice intravenously injected with 2 × 10^{12} vg AAV9 or MyoAAV4A. Scale bar = 2 mm. (**C**) Quantification of the direct GFP fluorescence in mouse hearts, n =5-6. Data are presented as mean ± SEM. Data were compared using Student's t-test. ***P* < 0.01. **; denotes comparison between MyoAAV4A and AAV9.

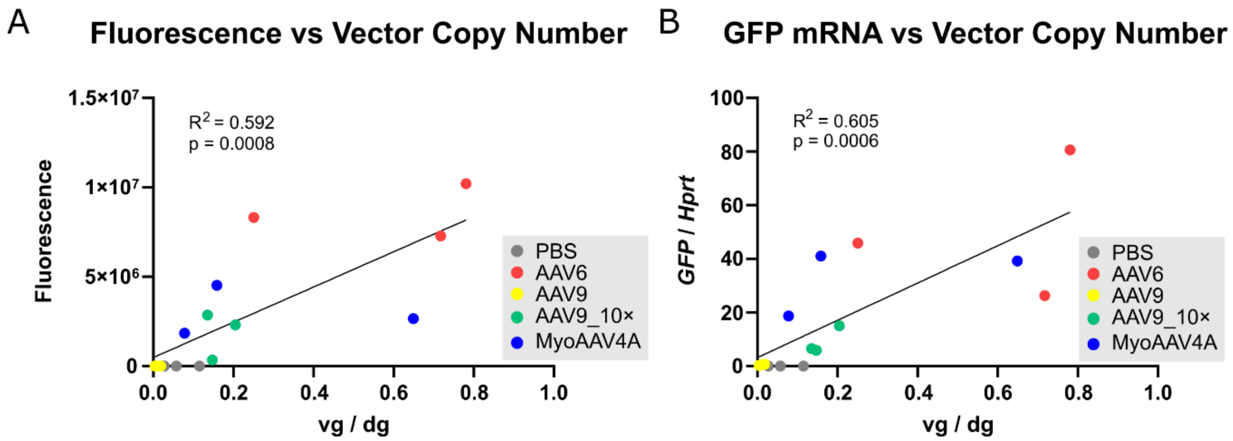

Figure S2. GFP expression correlates to AAV transduction in heart of mice intramyocardially injected with AAV-cTnT-GFP vectors.

(**A**) Correlation of GFP fluorescence and AAV vector copy number, n = 3. (**B**) Correlation of GFP mRNA expression level and AAV vector copy number, n = 3. Linear regression is shown in solid black lines. vg/dg, vector genomes per diploid genome.

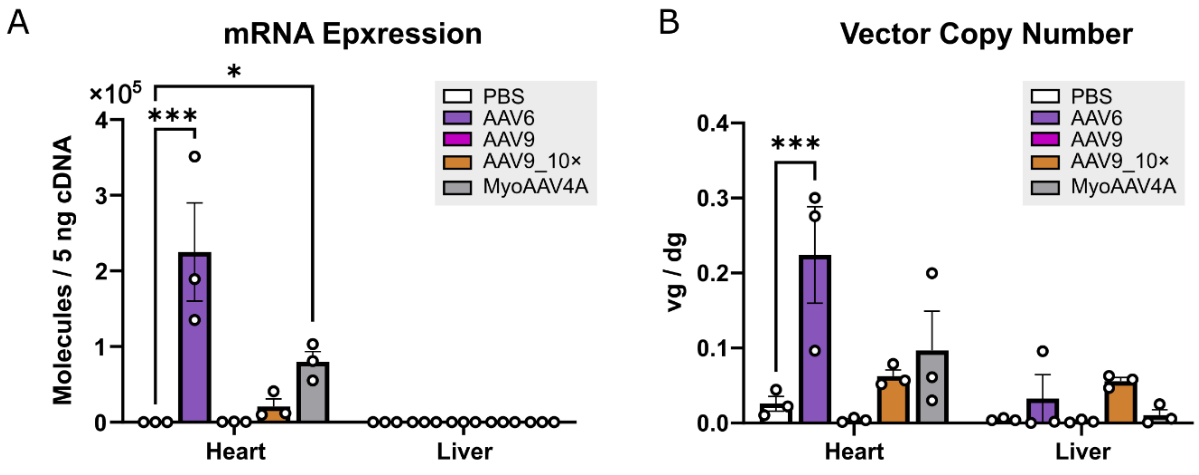

Figure S3. Intramyocardial injection of AAV-cTnT-GFP in mice does not lead to detectable transgene expression in the liver.

(**A**) mRNA expression level of GFP in heart and liver of mice intramyocardially injected with AAV-cTnT-GFP vectors, n = 3. (**B**) AAV genome copies in heart and liver of mice injected with AAV-cTnT-GFP vectors, n = 3. Data are presented as mean ± SEM. Data were compared using one-way ANOVA with *post-hoc* Fisher's LSD test. **P* < 0.05; ****P* < 0.001; ns, not significant. *; denotes comparison between groups and PBS. vg/dg, vector genomes per diploid genome.

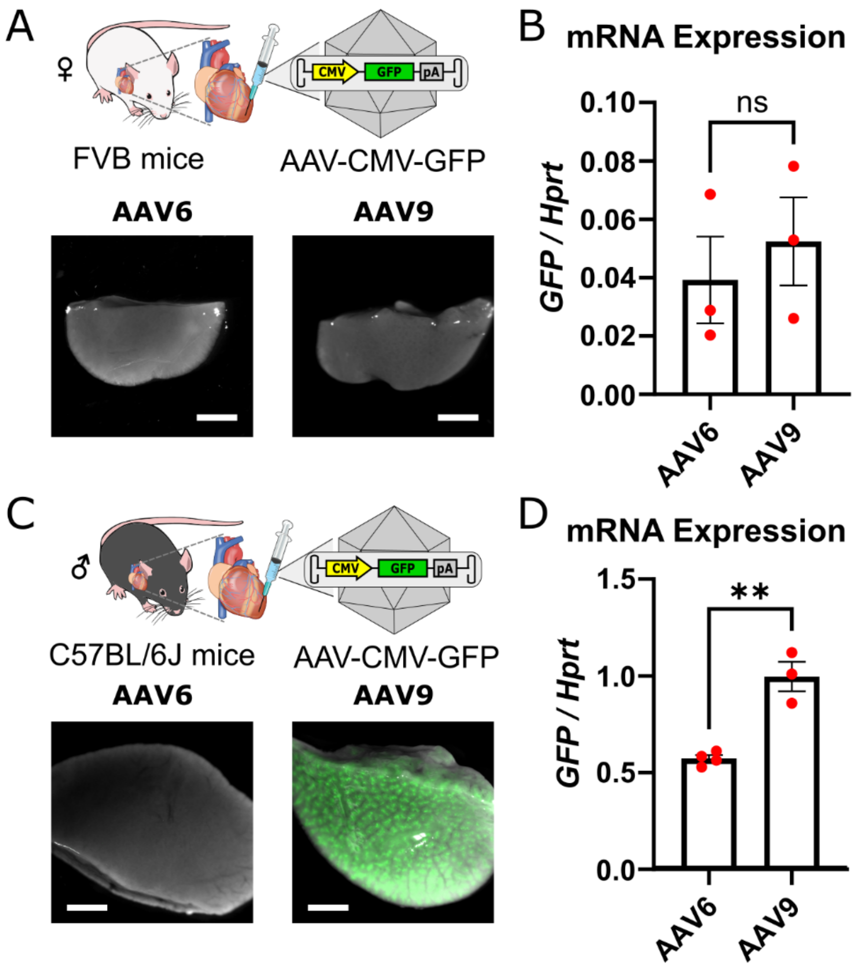

Figure S4. Intramyocardial injection of AAV9-CMV-GFP leads to higher liver expression in C57BL/6J mice than in FVB mice.

(A) Representative liver images of female FVB mice receiving direct intramyocardial injections with GFP-encoding AAV6 or AAV9 pseudotype vectors. Scale bar = 2 mm. (B) Quantification of GFP mRNA expression in the liver of female FVB mice, n = 3. (C) Typical liver images of male C57BL/B6J mice receiving direct intramyocardial injections with GFP-encoding AAV6 or AAV9 pseudotype vectors. Scale bar = 2 mm. (D) Quantification of GFP mRNA expression in the liver of male C57BL/6 mice, n = 3. Data are presented as mean ± SEM. Data were compared using Student's t-test. **P < 0.01; ns, not significant. **; denotes comparison between AAV6 and AAV9.

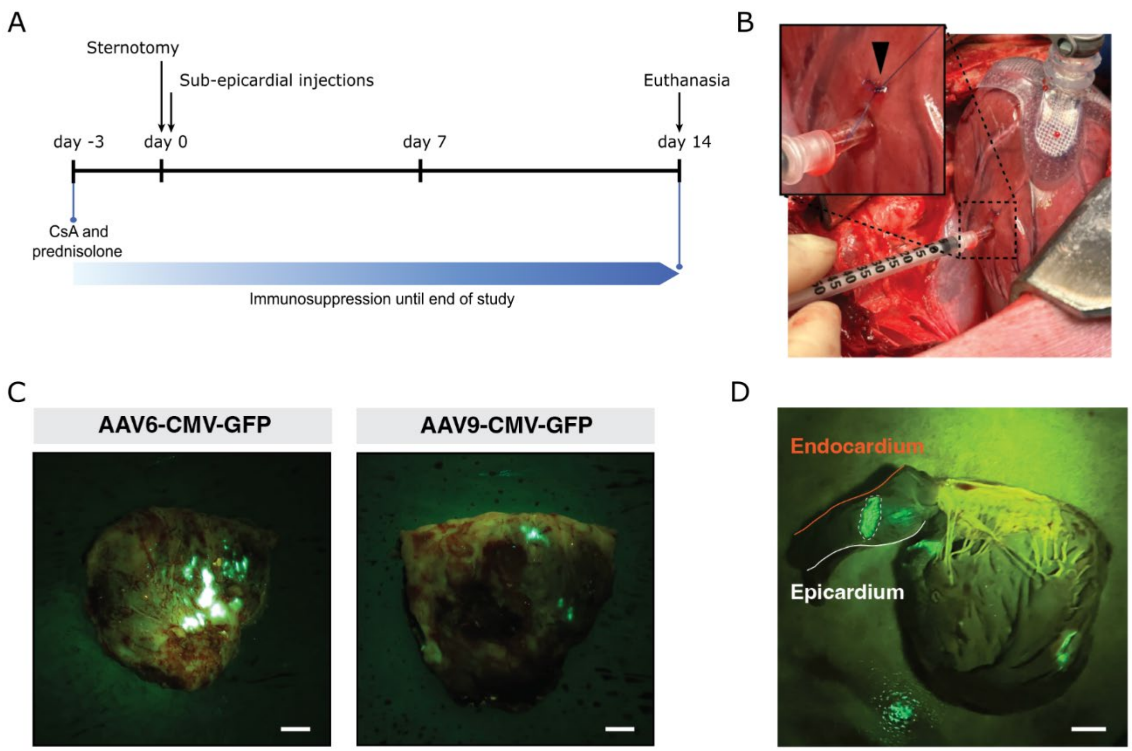

Figure S5. AAV vector-mediated GFP transduction in immunosuppressed pigs.

(A) Study timeline. (B) Heart stabilization from the apex using a heart positioner (Starfish™) to facilitate marking of the injection region with a suture (black arrow). (C) Epicardial view of GFP fluorescent areas from dissected left ventricles, 14 days post-transduction. Scale bar = 1 cm. (D) Endocardial dissection of the left ventricle of an AAV6-CMV-GFP-transduced pig displaying transmural transduction (white dashed oval). Scale bar = 1 cm.
